# Two-photon exchange in (muonic) deuterium at N3LO in pionless effective field theory

**DOI:** 10.1140/epja/s10050-022-00854-z

**Published:** 2022-11-16

**Authors:** Vadim Lensky, Franziska Hagelstein, Vladimir Pascalutsa

**Affiliations:** 1 Institut für Kernphysik, Johannes Gutenberg-Universität Mainz, 55128 Mainz, Germany; 2 Paul Scherrer Institut (PSI), 5232 Villigen, Switzerland

## Abstract

We present a study of the two-photon-exchange (2$\upgamma$-exchange) corrections to the *S*-levels in muonic ($\mu$D) and ordinary (D) deuterium within the pionless effective field theory (EFT). Our calculation proceeds up to next-to-next-to-next-to-leading order (N3LO) in the EFT expansion. The only unknown low-energy constant entering the calculation at this order corresponds to the coupling of a longitudinal photon to the nucleon–nucleon system. To minimise its correlation with the deuteron charge radius, it is extracted using the information about the hydrogen–deuterium isotope shift. We find the elastic 2$\upgamma$-exchange contribution in $\mu$D larger by several standard deviations than obtained in other recent calculations. This discrepancy ameliorates the mismatch between theory and experiment on the size of 2$\upgamma$-exchange effects, and is attributed to the properties of the deuteron elastic charge form factor parametrisation used to evaluate the elastic contribution. We identify a correlation between the deuteron charge and Friar radii, which can help one to judge how well a form factor parametrisation describes the low-virtuality properties of the deuteron. We also evaluate the higher-order 2$\upgamma$-exchange contributions in $\mu$D, generated by the single-nucleon structure and expected to be the most important terms beyond N3LO. The uncertainty of the theoretical result is dominated by the truncation of the EFT series and is quantified using a Bayesian approach. The resulting extractions of the deuteron charge radius from the $\mu$D Lamb shift, the $2S{-}1S$ transition in D, and the $2S{-}1S$ hydrogen–deuterium isotope shift, with the respective 2$\upgamma$-exchange effects evaluated in a unified EFT approach, are in perfect agreement.

## Introduction

Laser spectroscopy of muonic hydrogen ($\mu$H) and deuterium ($\mu$D) by the CREMA Collaboration in 2010, 2013 and 2016 enabled determinations of the proton and deuteron charge radii with unprecedented precision:

1a $$\begin{aligned} r_p(\mu \text {H})= & {} 0.84087(26)_\text {exp}(29)_\text {theory}\ \text {fm}\nonumber \\= & {} 0.84087(39)\ \text {fm [1,2]}, \end{aligned}$$

1b $$\begin{aligned} r_d(\mu \text {D})= & {} 2.12562(13)_\text {exp}(77)_\text {theory}\ \text {fm}\nonumber \\= & {} 2.12562(78)\ \text {fm [3]}, \end{aligned}$$

while the most accurate extraction of the deuteron charge radius,

2 $$ \begin{aligned} r_d(\mu \text {H }  \&  \text { iso})=2.12771(22)\ \text {fm}, \end{aligned}$$

is an indirect achievement combining measurements from the spectroscopy of ordinary and muonic atoms [2]: the 2*S*–1*S* hydrogen–deuterium (H–D) isotope shift and the Lamb shift in $\mu$H. This result is driving the presently recommended value of the deuteron charge radius from the CODATA 2018 report [4]:

3 $$\begin{aligned} r_d(\text {CODATA '18})=2.12799(74)\,\textrm{fm}. \end{aligned}$$

As one can see from Eq. (1), the charge radius extractions are limited by the theory uncertainty, which for muonic atoms is almost solely due to subleading nuclear-structure effects, and in particular, the $O(\alpha ^5)$ two-photon exchange (2$\upgamma$ exchange) discussed in this work.

The initial tension between the $r_d(\mu \text {D})$ and $r_d(\mu \text {H }  \&  \text { iso})$ extractions, shown above, was resolved in 2019 by amending the $\mu$D theory [5] to include the subleading $O(\alpha ^6)$ electronic vacuum polarization (VP) effects [6] and the inelastic three-photon exchange ($3\gamma$ exchange) [7]:

4 $$\begin{aligned} r_d(\mu \text {D})= & {} 2.12710(13)_\text {exp}(81)_\text {theory}\,\,\text {fm}\nonumber \\= & {} 2.12710(82)\ \text {fm [6]}. \end{aligned}$$

The deuteron-radius extractions from deuterium spectroscopy and electron–deuteron (*ed*) scattering are less precise and lead to larger values:

5a $$\begin{aligned} r_d(\text {D spectroscopy})= & {} 2.1415(45)\ \text {fm [8]}, \end{aligned}$$

5b $$\begin{aligned} r_d(ed\text { scattering})= & {} 2.130(10)\ \text {fm [9]}, \end{aligned}$$

5c $$\begin{aligned} r_d(\text {CODATA '14})= & {} 2.1413(25)\ \text {fm [10]}. \end{aligned}$$

This distinct discrepancy for the deuteron radius – the “deuteron radius puzzle” – is strongly affected by the 2$\upgamma$ exchange. It is thus timely to re-evaluate the 2$\upgamma$-exchange effects in a model-independent manner and try to improve their precision. While the latest developments [6, 7] are certainly important, they do not provide a path to a more systematic improvement of the theory error on the side of nuclear structure.

In this work, we consider the forward 2$\upgamma$-exchange contributions to D and $\mu$D, including the accompanying electronic VP contributions, within the pionless effective field theory (EFT) of nuclear forces [11–17]. This framework allows one to represent the nuclear observables in a well-defined perturbation theory, expanding in powers of the small parameter $P/m_\pi$, where *P* is the typical momentum scale (e.g., the size of the relative momentum between two nucleons, or that of the momentum of an external probe) and $m_\pi \simeq 139$ MeV is the pion mass. The typical momentum scale in the deuteron is characterized by the binding momentum $\gamma = \sqrt{M_N B}\simeq 45$ MeV, where $M_N$ is the nucleon mass and *B* is the deuteron binding energy. The momentum scale probed by the electromagnetic interaction in $\mu$D is $\sim \alpha m_\mu$, which is less than an MeV. This is also well below the limiting scale of the theory set by $m_\pi$. The atomic systems should thus be well-suited for the application of EFT. In addition, it has been shown that EFT provides a good description of low-energy experimental data on real deuteron Compton scattering [18, 19], and can be used to investigate the deuteron electric polarizability and electromagnetic form factors (FFs) [15]. Finally, the effective-field-theory (EFT) expansion allows one to quantify the theoretical uncertainty using methods such as Bayesian inference [20]. The basis for our 2$\upgamma$-exchange calculation is provided in Ref. [21], where closed analytic expressions for the unpolarized amplitudes of forward doubly-virtual Compton scattering (VVCS) off the deuteron are derived.

The paper is organized as follows. In Sect. 2, we briefly introduce the 2$\upgamma$-exchange and EFT frameworks. In Sect. 3, we calculate the elastic finite-size, inelastic deuteron-polarizability and single-nucleon contributions to the $\mu$D Lamb shift, and compare our results to other recent predictions. In Sect. 4, we repeat the same calculation for D and use the H–D isotope shift to fix the unknown low-energy constant (LEC) $l_1^{C0_S}$ that enters the VVCS amplitude. In Sect. 5, we utilize the unique possibility to cross-check the theoretical predictions for the 2$\upgamma$ exchange in $\mu$D with an empirical determination. The latter is extracted from the measured $\mu$D Lamb shift by fixing the deuteron charge radius to the independent value $r_d(\mu \text {H }  \&  \text { iso})$. We also compile an update for the theory prediction of the $\mu$D Lamb shift that will be used to extract $r_d(\mu \text {D})$ from the measurement of the CREMA Collaboration. In Sect. 6, we discuss deuteron and proton charge radii extractions from $\mu$D, D and the H–D isotope shift. A discussion of the neutron charge radius is postponed to Appendix G. In Sect. 7, we finish with conclusion and outlook. The appendices cover: (A) the Bayesian error analysis, (B) the inclusion of nucleon FFs beyond the EFT framework, (C) electronic VP corrections, and updated theory compilations for the: (D) $2S{-}1S$ H–D isotope shift, (E) $2S{-}1S$ in H, and (F) $2S{-}1S$ in D. Appendix E also has a determination of the Rydberg constant $R_\infty$ from $2S{-}1S$ in H and the Lamb shift in $\mu$H. A concise summary of our main results and their implications is published in Ref. [22].

**Fig. 1:**
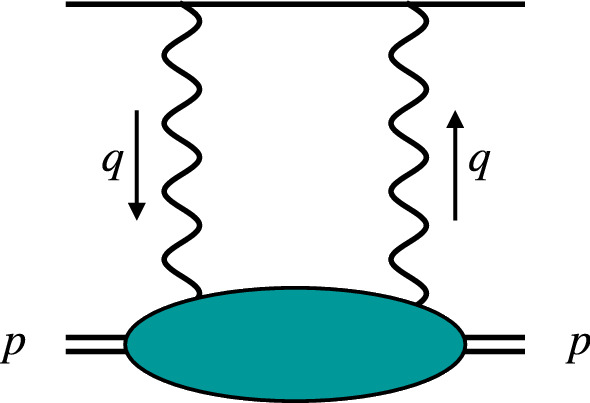
The leading order in $\alpha$ 2$\upgamma$-exchange potential

## Theoretical framework

### 2$\upgamma$ exchange in (muonic) deuterium

The leading order (LO) in $\alpha$ 2$\upgamma$-exchange correction corresponds to the forward kinematics, shown in Fig. 1. It gives a $\delta ({\varvec{r}})$-function correction to the Coulomb potential, thus, only shifts the *S*-levels, which have a non-vanishing atomic wave function at the origin. The spin-independent forward 2$\upgamma$ exchange is related to the VVCS amplitude off an unpolarized deuteron:

6 $$\begin{aligned} T_{fi} = \varepsilon _0\,\varepsilon _0^{\,\prime *}\,f_{L}(\nu ,Q^2) +(\varvec{\varepsilon } \cdot \varvec{\varepsilon }^{\,\, \prime *}) \,f_{T}(\nu ,Q^2), \end{aligned}$$

where $f_L(\nu ,Q^2)$ and $f_T(\nu ,Q^2)$ are the longitudinal and transverse scalar amplitudes with $Q^2=-q^2$ and $\nu = p\cdot q/M_d$ the photon virtuality and lab frame energy, and $M_d$ the deuteron mass. The modified photon polarization vector components are defined as

7 $$\begin{aligned} \varepsilon _0&=\left[ \epsilon _0-\frac{\nu }{\left| \varvec{q}\right| }\, (\varvec{\epsilon }\cdot {\hat{\varvec{q}}})\right] \frac{\left| \varvec{q}\right| }{Q},\nonumber \\ \varvec{\varepsilon }&= \varvec{\epsilon }-{\hat{\varvec{q}}}\,(\varvec{\epsilon }\cdot {\hat{\varvec{q}}}), \end{aligned}$$

with $\varvec{q}$ and $\varvec{\hat{q}}=\varvec{q}/|\varvec{q}|$ being the photon three-momentum in the lab frame and its unit vector, and $(\epsilon _0,\varvec{\epsilon })$ the time and space components of the photon polarization vector. This description in terms of $f_L(\nu ,Q^2)$ and $f_T(\nu ,Q^2)$ is natural for the EFT framework, but not unique. The explicitly covariant tensor decomposition with two other scalar amplitudes $T_i(\nu ,Q^2)$ related via

8 $$\begin{aligned} f_L(\nu ,Q^2)= & {} -T_1(\nu ,Q^2)+\left( 1+\frac{\nu ^2}{Q^2}\right) T_2(\nu ,Q^2), \nonumber \\ f_T(\nu ,Q^2)= & {} T_1(\nu ,Q^2), \end{aligned}$$

is widely used in, e.g., the dispersive 2$\upgamma$-exchange evaluations [23, 24]. We start from the covariant expression for the forward $O(\alpha ^5)$ 2$\upgamma$-exchange correction to the energy of a *nS* state in (muonic) deuterium, given in these references, and rewrite them in terms of the longitudinal and transverse amplitudes:

9a $$\begin{aligned} E_{nS}^\textrm{fwd}= & {} -8i\pi \alpha m \,\left[ \phi _{n}(0)\right] ^2\, \int \!\!\frac{\textrm{d}^4 q}{(2\pi )^4}\nonumber \\{} & {} \times \frac{\left( Q^2-2\nu ^2\right) T_1(\nu ,Q^2)-(Q^2+\nu ^2)\,T_2(\nu ,Q^2)}{Q^4(Q^4-4m^2\nu ^2)}\nonumber \\ \end{aligned}$$

9b $$\begin{aligned}= & {} 8i\pi \alpha m \,\left[ \phi _{n}(0)\right] ^2\, \int \!\!\frac{\textrm{d}^4 q}{(2\pi )^4}\nonumber \\{} & {} \times \frac{f_L(\nu ,Q^2)+2(\nu ^2/Q^2)f_T(\nu ,Q^2)}{Q^2(Q^4-4m^2\nu ^2)}, \end{aligned}$$

where *m* is the electron or muon mass, $[\phi _n(0)]^2=1/(\pi n^3 a^3)$ is the (Coulomb) wave function of the *nS* atomic state at the origin, $a=1/({\mathcal {Z}} \alpha m_r)$ is the Bohr radius, ${\mathcal {Z}}$ is the nuclear charge (${\mathcal {Z}}=1$ for the deuteron), and $m_r=m M_d/(m + M_d)$ is the atomic reduced mass. Separating the scalar amplitudes into the deuteron-pole and non-pole parts, one splits the 2$\upgamma$-exchange correction into the elastic and inelastic part [24]. The inelastic part, after doing the Wick rotation $\nu = iq_0$ and introducing the hyperspherical coordinates, takes the form:

10 $$\begin{aligned} E_{nS}^\textrm{inel}&=-\frac{\alpha }{2\pi ^2 m}\left[ \phi _{n}(0)\right] ^2 \int \limits _0^\infty \frac{\textrm{d}Q}{Q} \int \limits _{-1}^{1} \textrm{d}x\,\sqrt{1-x^2}\,\nonumber \\&\quad \times \frac{f_L(-iQx,Q^2)-2x^2 f_T(-iQx,Q^2)}{ \tau _l+x^2}, \end{aligned}$$

with $\tau _l=Q^2/(4m^2)$. Here we assume that the pole-part is subtracted from the scalar VVCS amplitudes. The elastic part of the 2$\upgamma$ exchange is readily obtained via the deuteron electromagnetic FFs – charge, magnetic, and quadrupole – $G_C(Q^2)$, $G_M(Q^2)$, and $G_Q(Q^2)$, resulting in [24]:

11 $$\begin{aligned} E_{nS}^\textrm{elastic}&= \frac{m \alpha ^2}{M_d(M_d^2-m^2)}[\phi _{n}(0)]^2 \int \limits _0^\infty 2\frac{\textrm{d}Q}{Q} \nonumber \\&\quad \times \left\{ \frac{2}{3}G_M^2(Q^2) (1+\tau _d){\hat{\gamma }}_1(\tau _d,\tau _l)\right. \nonumber \\&\quad -\left[ \frac{G_C^2(Q^2)-1}{\tau _d}+\frac{2}{3}G_M^2(Q^2) +\frac{8}{9}\tau _d G_Q^2(Q^2)\right] \nonumber \\&\quad \left. \times \,{\hat{\gamma }}_2(\tau _d,\tau _l) +16M_d^2\frac{M_d-m}{Q}G_C'(0) \right\} , \end{aligned}$$

where $\tau _d=Q^2/(4M_d^2)$, and the weighting functions are defined by:

12a $$\begin{aligned} {\hat{\gamma }}_{1,2}(x,y)&= \frac{\gamma _{1,2}(x)}{\sqrt{x}}-\frac{\gamma _{1,2}(y)}{\sqrt{y}}, \end{aligned}$$

12b $$\begin{aligned} \gamma _1(x)&= (1-2x)\sqrt{1+x}+2x^{3/2}, \end{aligned}$$

12c $$\begin{aligned} \gamma _2(x)&= (1+x)^{3/2}-x^{3/2} -\frac{3}{2}\sqrt{x}. \end{aligned}$$

Note that the contributions of point-like charge and charge radius of the deuteron are removed from the elastic part to avoid double counting [24]. This is done by subtracting the unity and the term proportional to $G_C'(0)$ in Eq. (11).

### Unpolarized deuteron VVCS in pionless EFT

In our analysis, we use results from the EFT calculation of the unpolarized deuteron VVCS amplitudes $f_L(\nu , Q^2)$ and $f_T(\nu ,Q^2)$ presented in Ref. [21]. This section gives a brief recap of the EFT framework applied to the deuteron VVCS, as well as a description of the technicalities relevant to the 2$\upgamma$-exchange calculation.

EFT is an EFT for nucleon interactions at low energies, where the high-energy scale is set by the pion mass $m_\pi$. If the momentum transfer between two nucleons is $P\ll m_\pi$, one can treat a pion-exchange interaction as a contact one. In EFT nucleons are thus interacting through contact interactions [11–13, 15]. The Lagrangian is constructed performing a non-relativistic expansion in the one-nucleon sector and writing out the relevant two-nucleon interactions [11–17]. To assign a particular order to a Feynman graph, one counts powers of momenta [$Q=O(P)$] and energies [$\nu =O(P^2)$] coming from the interaction vertices, nucleon propagators [$O(P^{-2})$], and loops [$O(P^5)$]. The small expansion parameter is the ratio $P/m_\pi$. For the deuteron, where the typical momentum scale is the binding momentum $\gamma$, this corresponds to $P/m_\pi \simeq 1/3$. Note that different momentum scales can count as different powers of the typical momentum *P*, depending on the problem setting. For instance, the counting we use has the photon three-momentum $|\varvec{q}|=O(P)$, whereas its energy is $\nu =O(P^2)$, and hence also its virtuality $Q=O(P)$. This reflects our expectation that the virtual photons in the $2\gamma$-exchange, as viewed in the lab frame, mostly transfer three-momentum, and very little energy, to the intermediate deuteron state, and is in contrast to, e.g., a typical real Compton scattering setting where $\nu =|\varvec{q}|$, implying they have to be of the same size in the counting.

Regarding the description of the deuteron state, one can use different prescriptions to perform the expansion around the deuteron pole of the nucleon–nucleon (*NN*) scattering amplitude. The *z*-parametrisation [17], chosen in Ref. [21, Sec. II B], is particularly well-suited for quantities such as the deuteron electric dipole polarizability $\alpha _{E1}$ that receive mostly long-range contributions and are thus sensitive to the correct description of the long-range tail of the deuteron wave function. This parametrisation reproduces the residue *Z* of the *NN* scattering amplitude at the deuteron pole at next-to-leading order (NLO). The residue is related to the effective range $\rho _d$ in the *NN* triplet channel via $Z=(1-\gamma \rho _d)^{-1}$, and is also connected to the asymptotic normalisation of the deuteron *S*-wave via

13 $$\begin{aligned} \psi (r)\xrightarrow {r\rightarrow \infty }\sqrt{\frac{\gamma Z}{2\pi }}\frac{e^{-\gamma r}}{r}. \end{aligned}$$

It is therefore straightforward to see that this procedure also reproduces the correct large-distance asymptotics at NLO. Note also that it introduces a new formal expansion parameter $(Z-1)=O(P)$.

Analysing the counting for the VVCS shows [21, Sec. II B] that the longitudinal amplitude, driven by the deuteron electric polarizability $\alpha _{E1}$, is dominant, starting at $O(P^{-2})$, whereas the transverse amplitude starts two orders higher at *O*(*P*). In the context of the 2$\upgamma$-exchange correction, Eq. (9b) shows that the $f_T$ contribution is additionally suppressed, compared to the contribution of $f_L$, by the factor $\nu ^2/Q^2 = O(P^2)$. The transverse contribution to the 2$\upgamma$-exchange correction therefore starts only at $O(P^2)$, or N4LO compared to the leading longitudinal contribution. It is also at N4LO that, as explained in Ref. [21], higher powers of momenta entering the EFT expansion render the 2$\upgamma$-exchange correction naively divergent. This divergence ought to be absorbed by a four-nucleon two-lepton contact term entering at this order, and, since there is no data that would allow one to pinpoint the corresponding coupling other than the 2$\upgamma$-exchange correction itself, this is where the predictive power of EFT is exhausted. This motivated us to calculate the longitudinal amplitude up to N3LO in Ref. [21]. We also calculated the transverse amplitude up to *O*(*P*), or its respective NLO; this allows us to quantify here the corresponding 2$\upgamma$-exchange contribution.

Further details of the EFT framework used to calculate $f_L$ and $f_T$ at their respective N3LO and NLO can be found in Ref. [21, Sec. II]. The results for the VVCS amplitudes are given in a closed analytic form in Ref. [21, Sec. III], in terms of the longitudinal and transverse four-point functions ${\mathcal {M}}_{L,T}(\nu ,Q^2)$ and the inverse of the derivative of the deuteron self-energy (SE) at the deuteron pole $\left[ \Sigma '(E_d)\right] ^{-1}$. We use them here to calculate the 2$\upgamma$-exchange correction. At N3LO in the EFT expansion of $f_L$, one encounters a previously undetermined coupling $l_1^{C0_S}$ of a longitudinal photon to the two-nucleon system, which contributes, in particular, to $G_C(Q^2)$ and $r_d$. The latter quantity was used in Ref. [21] to extract $l_1^{C0_S}$ from a fit to $r_d(\mu \text {D})$ in Eq. (1b). This procedure is potentially problematic due to the fact that $l_1^{C0_S}$ also enters the 2$\upgamma$-exchange correction, both in $\mu$D and the isotope shift. We investigate the resulting correlations below and demonstrate explicitly that they are negligible at the current level of theoretical and experimental precision.

## 2$\upgamma$ exchange in muonic deuterium

In the following section, we will present in detail our calculation of the elastic and inelastic 2$\upgamma$-exchange contributions to the Lamb shift in $\mu$D. A summary of our results can be found in Sect. 3.4.

### Elastic contribution

We start by considering the elastic contribution to the 2$\upgamma$-exchange correction based on the EFT deuteron FFs. Taking the N3LO result for $G_C(Q^2)$ in Ref. [21, Eq. (75)] and expanding in Eq. (11) also to N3LO results in

14 $$\begin{aligned} E_{2S}^\textrm{elastic}=\left[ -0.4482-0.9938\, l_1^{C0_S} \right] \text { meV}. \end{aligned}$$

This neglects the magnetic and quadrupole FFs, whose contributions are subleading in the EFT counting and are indeed numerically very small, see below in Table 2. The electric contact term coupling $l_1^{C0_S}$ can be fixed through the deuteron charge radius:

15 $$\begin{aligned} r_d^2\equiv - 6\, G_C'(0) = \frac{1}{8 \gamma ^2} +\frac{Z-1}{8 \gamma ^2} +2r_0^2 +\frac{3(Z-1)^3}{\gamma ^2}\,l_1^{C0_S}, \nonumber \\ \end{aligned}$$

with  being the isoscalar nucleon charge radius, with the proton Darwin–Foldy term  added to it. Previously, $l_1^{C0_S}$ was chosen to reproduce the deuteron charge radius from $\mu$D spectroscopy, resulting in [21]

16 $$\begin{aligned} l_1^{C0_S}=-2.32(41)\times 10^{-3}, \end{aligned}$$

where the uncertainty stems from the error of the deuteron radius, Eq. (1b), and the uncertainty of *Z*. However, the extraction of $r_d^2$ from $\mu$D spectroscopy depends on the theory result for the 2$\upgamma$-exchange correction (even though the contribution of $l_1^{C0_S}$ to the 2$\upgamma$-exchange correction is small). This correlation can be practically eliminated if the deuteron radius extracted from the combination of the proton radius and the H–D $2S{-}1S$ isotope shift, as given in Eq. (2), is used as the reference data point. One has to note that the isotope shift also has a 2$\upgamma$-exchange contribution, but its relative importance as well as its correlation with $r_d^2$ is much smaller. To investigate this issue quantitatively, we perform a re-analysis of the isotope shift using the EFT formalism to predict the 2$\upgamma$-exchange correction in ordinary D, see Sect. 4 and Appendix D. Our calculation confirms that the contribution of $l_1^{C0_S}$ to the isotope shift can indeed be safely neglected. The corresponding result for the electric contact term coupling, which will be used throughout this work, is

17 $$\begin{aligned} l_1^{C0_S}=-1.80(38)\times 10^{-3}. \end{aligned}$$

This agrees with the result that we deduced from Eq. (2) [25], but differs from Eq. (16) by about $1\, \sigma$, since the extraction via the isotope shift gives a value of $r_d(\mu \text {H }  \&  \text { iso})$ slightly different from $r_d(\mu \text {D})$ in Eq. (1b). The related effect on $E_{2S}^\textrm{elastic}$ is small. The final numerical result for the elastic contribution is:

18 $$\begin{aligned} E_{2S}^\textrm{elastic}&=[-0.2043-0.1582-0.0626-0.0213]~\textrm{meV}\nonumber \\&= -0.4463(77) \text { meV}, \end{aligned}$$

where the numbers here stand for the order-by-order contributions. The uncertainty of $E_{2S}^\textrm{elastic}$ is due to higher orders in the EFT expansion; we quantify it as explained in Appendix A.

**Table 1:** $E_{2S}^\textrm{elastic}$, $E_{2S}^{\textrm{inel},L}$ and their sum $E_{2S}^\textrm{sum}$ in detail: contributions appearing at each order in the expansion. Values are in meV. Upper indices indicate the order of ${\mathcal {M}}_L$ that generates the corresponding contribution, see Ref. [21]. Quantities without labels are the contributions at the respective order excluding the labelled terms listed separately. Labels indicate specific terms within ${\mathcal {M}}_L$: $r_N^2$, $w_2$, *P*, and $l_1^{C0_S}$ stand, in order, for the nucleon charge radii correction, the contribution proportional to the *NN* triplet *S*-wave shape parameter $w_2$, the *NN* *P*-wave contribution, and the contribution proportional to $l_1^{C0_S}$

	$E_{2S}^\textrm{elastic}$	$E_{2S}^{\textrm{inel},L}$	$E_{2S}^\textrm{sum}$
	LO		
$E^{(-3)}$	$-0.2043$	$-0.9433$	$-1.1476$
	NLO		
$(Z-1)E^{(-3)}$	$-0.1408$	$-0.6502$	$-0.7910$
$E^{(-2)}$	$-0.0174$	0.0153	$-0.0021$
	N2LO		
$(Z-1)E^{(-2)}$	$-0.0120$	0.0106	$-0.0014$
$E^{(-1)}$	0	$-0.0006$	$-0.0006$
$E^{(-1)}_{r_N^2}$	$-0.0506$	0.0389	$-0.0117$
	N3LO		
$(Z-1)E^{(-1)}$	0	$-0.0004$	$-0.0004$
$(Z-1)E^{(-1)}_{r_N^2}$	$-0.0349$	0.0268	$-0.0081$
$E^{(0)}$	0	$-0.0009$	$-0.0009$
$E^{(0)}_{w_2}$		0.0002	0.0002
$E^{(0)}_{P}$		0.0068	0.0068
$$ E^{(0)}_{l_1^{C0_S}}$$	0.0018	$$-0.0012$$	0.0006
$E^{(0)}_{r_N^2}$	0.0118	$-0.0063$	0.0055

To study the elastic (and inelastic) contribution in detail, we split them as shown in Table 1, keeping track of different terms appearing both due to the $(Z-1)$ factors coming from the NLO piece of $\left[ \Sigma ^\prime (E_d)\right] ^{-1}$ and due to new sources at each order in the longitudinal four-point function ${\mathcal {M}}_L$. This representation will also be useful below in the investigation of the theoretical uncertainty. In order to split the elastic term this way, it is convenient to rewrite the last term in Eq. (11) replacing $G_C'(0)$ by , where the normalization of $G_C(0)=1$ is used. This reflects the fact that the elastic part of the VVCS amplitude is proportional to the deuteron FFs squared, and allows one to separate the contributions in the integrand without generating spurious singularities at $Q=0$. One can see from the table that the most important contributions to $E_{2S}^\textrm{elastic}$ come, as expected, from the LO part of ${\mathcal {M}}_L$, with the nucleon charge radius contributions providing the most important correction at N2LO. One can also see that the only new contributions beyond NLO come either from the nucleon structure or from the N3LO contact term proportional to $l_1^{C0_S}$. The nucleon charge radius contributions may seem somewhat larger than expected at N2LO and N3LO; to judge whether this is an indication of potentially sizeable corrections to $E_{2S}^\textrm{elastic}$ beyond N3LO, it is instructive to look at the details of the deuteron charge FF at small photon virtualities. Indeed, it is evident from Eq. (11) that the 2$\upgamma$-exchange integrand is strongly weighted towards low $Q^2$. Therefore, it is the slopes and the curvatures of the deuteron FFs at $Q^2=0$ that will have significant influence on the elastic contribution. The slope of the charge FF, proportional to $r_d^2$, is reproduced at N3LO; based on that alone, a sizeable modification of the shape of $G_C(Q^2)$ at small $Q^2$ could come from higher-order coefficients in its expansion in powers of $Q^2$. To look into this issue, we review the calculation of $E_{2S}^\textrm{elastic}$ using several different deuteron FFs along with the N3LO EFT result, and investigate how the features of those FFs affect the result.

**Fig. 2:**
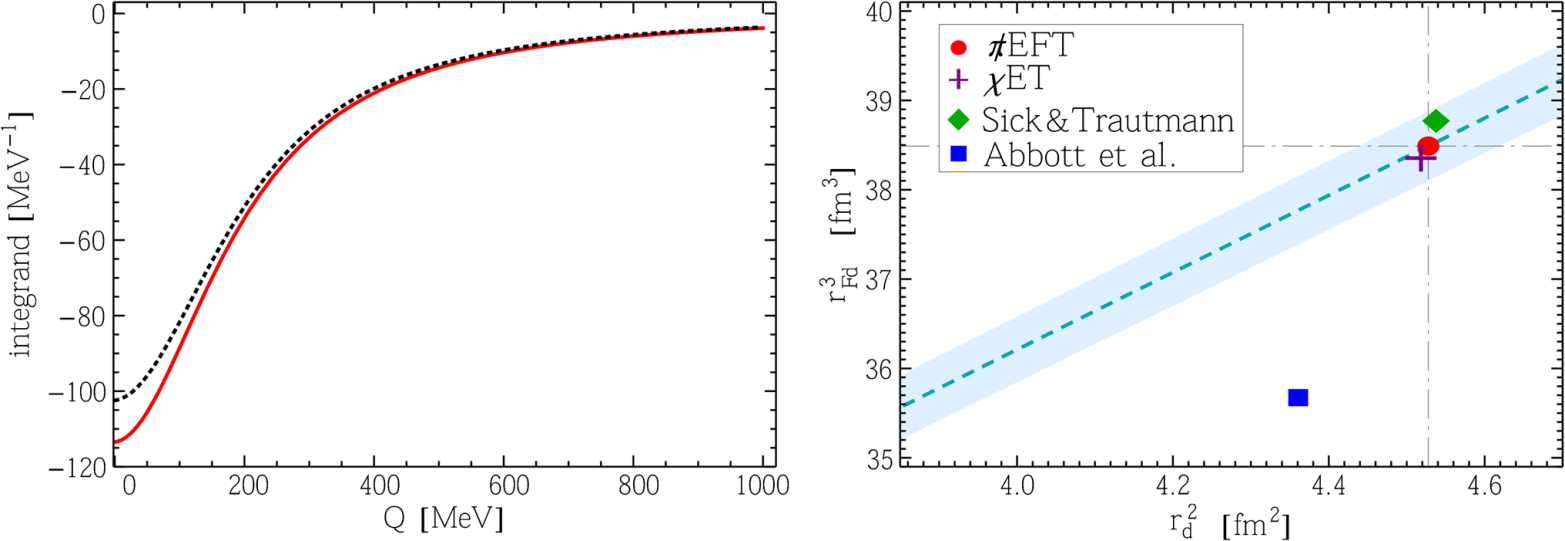
Left panel: integrand of Eq. (11) as function of *Q*. Black dotted: deuteron form factor parametrisations from Ref. [28]; red solid: result of the EFT calculation. Right panel: correlation of $r_{\textrm{F}d}^3$ and $r_d^2$. The dashed line shows the correlation obtained from the EFT results at N3LO, with the band showing the estimated $~1\%$ N3LO uncertainty; the red disc, purple cross, green diamond, and blue square show the values obtained, respectively, from EFT at N3LO, the N4LO $\chi$ET form factor [27], the parametrisation of Ref. [9], and the parametrisation of Ref. [28]

Starting with the recent higher-order, N4LO in the respective counting, chiral effective theory ($\chi$ET) calculation of Refs. [26, 27], a good agreement between the N3LO EFT and the N4LO $\chi$ET results for $G_C(Q^2)$ at low *Q* was pointed out in Ref. [21]. As expected, our result for $E_{2S}^\textrm{elastic}$ perfectly agrees with what one obtains using the $\chi$ET charge FF from Ref. [27]:

19 $$\begin{aligned} E_{2S}^\textrm{elastic}(\chi \text {ET})&= -0.4456(18)\text { meV}, \end{aligned}$$

where we neglected the magnetic and quadrupole contributions, and evaluated the uncertainty using the uncertainty of the $\chi$ET result for $G_C(Q^2)$. On the other hand, using the recent empirical deuteron FFs from Ref. [28], Carlson et al. obtained a considerably smaller value [24]:

20 $$\begin{aligned} E_{2S}^\textrm{elastic}(\text {emp. FF [28]})&= -0.417(2) \text { meV}, \end{aligned}$$

with the uncertainty estimated using the different FF parametrisations derived in [28]. The same result has been adopted in Ref. [29]. We repeat this calculation, separating the contributions from the charge, magnetic and quadrupole FFs. The results are presented in Table 2 (using parametrisation II of Abbott et al.), along with values obtained by us based on the Sick and Trautmann parametrisation [9], as well as the $\chi$ET and EFT FFs. One can see that the contributions of both the magnetic and quadrupole FFs to the elastic part of the 2$\upgamma$-exchange correction can be safely neglected at the current level of precision. While the values of $E_{2S}^\textrm{elastic}$ obtained in EFT, $\chi$ET, and with the Sick and Trautmann parametrisation of the deuteron FF agree, the parametrisation of Abbott et al. gives a significantly smaller value for the elastic contribution. The left panel of Fig. 2 shows that this discrepancy is due to the behaviour of the parametrisation of Ref. [28] being very different from the other three calculations (which would all overlap) at low *Q*.

**Table 2:** Deuteron form factor contributions to the elastic 2$\upgamma$ exchange. Values are in meV. The magnetic and quadrupole contributions are omitted in the $\chi$ET calculation. In EFT, those contributions first start at N4LO

Deuteron form factor	$G_C$	$G_M$	$G_Q$	$E_{2S}^\textrm{elastic}$
Abbott et al. [28]	$-0.4153$	$<10^{-4}$	$-0.0007$	$-0.417(2)$ [24]
Sick and Trautmann [9]	$$-0.4503 $$	$$<10^{-4}$$	$$-0.0006$$	$$-0.4509$$
$\chi$ET N4LO [27]	$-0.4456(18)$	/	/	$-0.4456(18)$
EFT N3LO	$-0.4463(77)$	0	0	$-0.4463(77)$

Expanding the integrand in Eq. (11) at small *Q* [neglecting the $G_M(Q^2)$ and $G_Q(Q^2)$ contributions], one obtains

21 $$\begin{aligned}&\frac{2}{Q}\left[ -\frac{G_C^2(Q^2)-1}{\tau _d}{\hat{\gamma }}_2(\tau _d,\tau _l) +16M_d^2\frac{M_d-m}{Q}G_C'(0)\right] \nonumber \\&\quad = 4 M_d (M_d-m) \Big [4 M_d\, G_C''(0)-4 M_d\, G_C'(0)^2 \nonumber \\&\qquad +3 G_C'(0)/m\Big ] +O(Q). \end{aligned}$$

Therefore, the bulk of the difference can be further traced down to the deuteron charge radius and the $4{\textrm{th}}$ moment of the deuteron charge density: $G_C'(0)=-r_d^2/6$ and $G_C''(0)=\big <r^4_d\big >/60$. Also interesting are two further quantities related to the elastic 2$\upgamma$-exchange contribution, namely, the cubic and the Friar radii, defined respectively as [30]:

22a $$\begin{aligned} \big <r^3_d\big >&= \frac{48}{\pi }\int \limits _0^\infty \frac{\textrm{d}Q}{Q^4}\left[ G_C(Q^2)-1-G'_C(0)\,Q^2\right] , \end{aligned}$$

22b $$\begin{aligned} r_{\textrm{F}d}^3&= \frac{48}{\pi }\int \limits _0^\infty \frac{\textrm{d}Q}{Q^4}\left[ G_C^2(Q^2)-1-2G'_C(0)\,Q^2\right] . \end{aligned}$$

In EFT at N3LO, the considered moments have the following analytic expressions, obtained using $G_C(Q^2)$ from Ref. [21, Eq. (75)] (note again that the integrand in $r_{\textrm{F}d}^3$ has to be expanded up to N3LO):

23a $$\begin{aligned} r_{\textrm{F}d}^3&= \frac{3}{80\gamma ^3}\Bigg \{ Z \left[ 5 - 2 Z (1-2\ln 2)\right] \nonumber \\&\quad -\frac{320}{9} r_0^2\gamma ^2 \left[ Z(1-4\ln 2)-2+2\ln 2\right] \nonumber \\&\quad + 80 (Z-1)^3\, l_1^{C0_S}\Bigg \}, \end{aligned}$$

23b $$\begin{aligned} \big <r^3_d\big >&= \frac{Z}{32\gamma ^3}\left( 3+32\,r_0^2\gamma ^2\right) , \end{aligned}$$

23c $$\begin{aligned} \big <r^4_d\big >&=\frac{Z}{96\gamma ^4 }\left( 9+80\, r_0^2 \gamma ^2\right) . \end{aligned}$$

Table 3 shows the values of these quantities for the considered FFs. It is evident that parametrisation II of Abbott et al. [28] gives smaller values for all radii. Smaller $r_d$ and $\left\langle r_d^4\right\rangle$ lead to a significantly smaller value of the integrand at low *Q*, as seen in the left panel of Fig. 2, and consequently a smaller $E_{2S}^\textrm{elastic}$ as well as smaller Friar and cubic radii. Note that, neglecting recoil corrections, the elastic contribution can be approximated through the Friar radius as [30]

24 $$\begin{aligned} E^\mathrm {elastic,\ F}_{2S}&=-\frac{m_r^4\alpha ^5}{24} r_{\textrm{F}d}^3. \end{aligned}$$

This approximation, however, results in a noticeable underestimation of $E_{2S}^\textrm{elastic}$. The EFT value, for instance, turns out to be $E_{2S}^\mathrm {elastic,\ F} = -0.4323 \text { meV}$, which has to be compared to Eq. (18). We therefore conclude that at the present level of theoretical precision it is important to retain the full weighting function ${\hat{\gamma }}_2(\tau _d,\tau _l)$ in Eq. (11) instead of only taking the leading Friar radius term.

**Table 3:** Various radii corresponding to the different deuteron charge form factors

Radii [fm]	EFT N3LO	$\chi$ET N4LO [27]	Sick and Trautmann [9]	Abbott et al. [28]
$r_d$	2.128	2.126	2.130(10)	2.094(9)
$r_{\textrm{F}d}$	3.376	3.372	3.385	3.292
	2.468	2.468	2.480	2.401
	2.820	2.837	2.844	2.726

The dependence of both $r_d^2$ and $r_{\textrm{F}d}^3$ on $l_1^{C0_S}$ can be represented as a linear correlation between these quantities. We show the correlation line in the right panel of Fig. 2, where we also plot a $\pm 1\%\sim (\gamma /m_\pi )^4$ band as a simple estimate of terms beyond N3LO in the EFT expansion. One can see that the N4LO $\chi$ET result lies almost on the correlation line, very close to the EFT results fixed by the H–D $2S{-}1S$ isotope shift, see Sect. 4 and Appendix D. The parametrisation of Ref. [9] lies some distance from the line, albeit reasonably close to it, whereas that of Ref. [28] is much further away. It would be interesting to see if this correlation line can be reproduced in a $\chi$ET calculation.

The above considerations indicate that the FF parametrisation of Ref. [28], used in Refs. [24, 29], might not adequately describe the behaviour of the deuteron charge FF at low virtualities. The agreement between the N3LO EFT and N4LO $\chi$ET calculations, see Ref. [21, Sec. IV] for a detailed comparison of the FFs, is not entirely surprising as both these EFTs are expected to well reproduce low-momenta/long-range properties of the deuteron, and both calculations are of sufficiently high orders in the respective expansions. This vindicates our choice of the EFT as the calculational framework. One can also conclude that the correlation shown in Fig. 2 can serve as a diagnostic criterion for a realistic parametrisation of the deuteron charge FF. Furthermore, one can note that the EFT expression for the deuteron charge FF at N3LO, as given in Ref. [21, Sec. IV], can serve as an analytic one-parameter fit to the electron–deuteron scattering data in the low-$Q^2$ range that is to be covered in the planned DRad experiment [31].

### Inelastic contribution

The calculation of the longitudinal contribution to inelastic part of the 2$\upgamma$-exchange correction with the known $f_L(\nu ,Q^2)$ is straightforward. The only technical complication is that the longitudinal term in the integral of Eq. (10) goes as $f_L(0,Q^2)/Q^3\propto 1/Q$ for $Q\rightarrow 0$ when one sets $x=0$. This singularity, however, is spurious, and can be avoided by subtracting from $f_L(\nu ,Q^2)$ its static part:

25 $$\begin{aligned} f_L(\nu ,Q^2)&= f_L(0,Q^2) + \left[ f_L(\nu ,Q^2)-f_L(0,Q^2)\right] . \end{aligned}$$

The integration over *x* in the integral of $f_L(0,Q^2)$ can be done analytically, resulting in a $f_L(0,Q^2)/Q^2\propto Q^0$ behaviour for $Q\rightarrow 0$. At the same time, the difference in the square brackets is $O(x^2)$ at small *x* and therefore cancels the singularity in the weighting function. The longitudinal contribution then results in:

26 $$\begin{aligned} E_{2S}^{\textrm{inel},L} = \left[ -1.5032 + 0.6350\, l_1^{C0_S}\right] \ \text {meV}. \end{aligned}$$

One can see that the coefficients in front of $l_1^{C0_S}$ here and in Eq. (14) partially cancel each other. The resulting contribution of the N3LO contact term to the 2$\upgamma$ exchange in $\mu$D is rather small. The numerical order-by-order result for $E_{2S}^{\textrm{inel},L}$, using $l_1^{C0_S}$ as obtained from the H–D isotope shift, is:

27 $$\begin{aligned} E_{2S}^{\textrm{inel},L}= & {} [-0.943 - 0.635 + 0.049 + 0.025]~\textrm{meV} \nonumber \\= & {} - 1.504(16) \ \text {meV}. \end{aligned}$$

The uncertainty here is due to higher-order terms in the EFT expansion, calculated as explained in Appendix A. The individual terms of the inelastic contribution are shown in Table 1, in an analogy to what is shown for the elastic part. While the bulk of $E_{2S}^{\textrm{inel},L}$ is given by the LO part of ${\mathcal {M}}_L$, the most important correction comes from the nucleon charge radii, with the second-biggest correction driven by the NLO term of ${\mathcal {M}}_L$. The remaining mechanisms all give much smaller contributions.

The above results, Eqs. (26), (27), and Table 1, are obtained with the substitution $|\varvec{q}|\rightarrow Q$ in the expressions for ${\mathcal {M}}_L$; using $|\varvec{q}|=\sqrt{Q^2+\nu ^2}$ brings the total value to $-1.507$ meV. This gives an estimate of the relativistic corrections at N4LO. The smallness of the effect corroborates the choice of counting scheme in our calculation, namely, that the energy transfer is suppressed and $\nu /Q=O(P)$. This statement can be made more quantitative by observing that shrinking the *x* integration interval in Eq. (10) to  retains $\sim 96\%$ of the LO$+$NLO contribution. Furthermore, the transverse contribution to $E_{2S}^\textrm{inel}$, calculated at NLO for $f_T(\nu ,Q^2)$, is small in accordance with the prediction of the EFT counting:

28 $$\begin{aligned} E_{2S}^{\textrm{inel},T}=-0.005\text { meV}. \end{aligned}$$

It is also in a very good agreement with the existing dispersive $\chi$ET-based evaluations [29, 32]. Despite the smallness of the transverse contribution, we add it to the total inelastic contribution, since it is included in most of the alternative calculations, thus having

29 $$\begin{aligned} E_{2S}^\textrm{inel}=E_{2S}^{\textrm{inel},L}+E_{2S}^{\textrm{inel},T}=-1.509(16)\,\textrm{meV}. \end{aligned}$$

The uncertainty of the transverse contribution is neglected.

Based on the observations above, we conclude that the EFT counting used by us works well for the present calculation. We also do not expect any higher-order corrections that would change the pattern that one sees at N3LO; a quantification of this statement follows through the Bayesian procedure in Appendix A.

In Table 4, we compare our $E_{2S}^\textrm{inel}$ result with other recent evaluations. Our result agrees with the recent covariant dispersive calculation [29] as well as with the value quoted in Ref. [32] within the uncertainties. The latter has a slightly larger in magnitude central value. These two results obtain the deuteron response functions at N3LO in the $\chi$ET expansion to calculate $E_{2S}^\textrm{inel}$ from a dispersive integral. The data-driven evaluation of Carlson et al. [24] also uses a dispersive approach, but extracts information on the deuteron response functions from experimental data. It calculates an even larger $E_{2S}^\textrm{inel}$ with a large uncertainty that makes it compatible with all other results. In addition, we compare the results in the point-nucleon limit, where the contributions from the nucleon charge radii are removed (in which case we also omit the contribution of $l_1^{C0_S}$). Our result here is compatible with the earlier N3LO $\chi$ET result [32], as well as with that obtained from the N2LO EFT deuteron longitudinal response function in the point-nucleon limit [33].

**Table 4:** Comparison of our results with other recent calculations for the inelastic contribution $E_{2S}^\textrm{inel}$ and the inelastic contribution in the point-nucleon limit $E_{2S}^\mathrm {inel,\ p.N.}$. The latter is the inelastic contribution with point-like nucleons (calculated up to N3LO, with the contribution of $l_1^{C0_S}$ omitted). Values are in meV. To compare with Ref. [32], we subtract the subleading $O(\alpha ^6\log \alpha )$ Coulomb correction from their “$\eta$-less” result. The uncertainty given here for their prediction is obtained using the relative uncertainties of individual error sources from Ref. [34, Table 8] (nuclear model, isospin symmetry breaking, relativistic, higher ${\mathcal {Z}}\alpha$) summed in quadrature. The value quoted for Ref. [33] is their “${\mathcal {Z}}_d$-improved” result

	EFT N3LO	Acharya et al. [29]	Hernandez et al. [32]	Emmons et al. [33]	Carlson et al. [24]
$E_{2S}^\textrm{inel}$	$-1.509(16)$	$-1.511(12)$	$-1.531(12)$		$-1.566(740)$
$E_{2S}^\mathrm {inel,\ p.N.}$	$-1.567$		$-1.571$	$-1.574(80)$	

### Single-nucleon effects beyond N3LO

The results of Sects. 3.1 and 3.2 show that single-nucleon contributions generated by the hadron structure, such as the nucleon FFs, are the most important corrections beyond the LO and NLO nuclear-structure effects. They are also potentially the most problematic, since they tend to be enhanced by factors of $\varvec{q}^2$ compared with the corresponding amplitude with point-like nucleons. For instance, an N4LO correction with two insertions of the nucleon charge radius operator in the LO ${\mathcal {M}}_L$ diagrams, shown in Ref. [21, Fig. 7], would be enhanced by a factor of $\varvec{q}^4$ and would lead to a contribution to $E_{2S}$ that is divergent at large *Q*. Another potentially sizeable single-nucleon effect, first appearing also at N4LO, is that of the nucleon polarizabilities. Their inclusion into the deuteron VVCS amplitude also leads to a similar divergent contribution. A EFT consideration would therefore introduce four-nucleon and two-lepton contact terms at N4LO to regularise the divergence generated by the single-nucleon terms. Those contact terms, as pointed out in Sect. 2.2, limit the predictive powers of EFT in the study of the 2$\upgamma$-exchange corrections to N3LO. In this section, we quantify these hadron structure effects, expected to be the most important ones beyond N3LO, using alternative methods that go beyond the EFT expansion.

Starting from the nucleon FF, one alternative that can improve the bad behaviour of the nucleon FF correction would be to insert the full nucleon FFs in the nucleon charge operator vertex, replacing its LO term according to:

30 $$\begin{aligned} \frac{1}{2}(1+\tau _3)\rightarrow \frac{\hat{G}^N_E(Q^2)}{\sqrt{1+\frac{Q^2}{4M_p^2}}}, \end{aligned}$$

where $\hat{G}^N_E(Q^2)=G_E^{0}(Q^2)+G_E^1(Q^2)\,\tau _3$ with $G_E^{0,1}$ being the isoscalar and isovector nucleon electric FFs, $G_E^{0,1}(Q^2)=\left[ G_E^p(Q^2)\pm G_E^n(Q^2)\right] /2$. This procedure obviously represents a departure from the strict EFT treatment. It provides, however, a viable workaround and allows one to estimate the effects generated by the higher-order terms in the expansion of the nucleon FFs. It also is routinely used in $\chi$ET calculations of electromagnetic processes in nuclei, since the nucleon FFs do not converge well in a chiral expansion, either, see Refs. [27, 29] for recent examples. The specific substitution of Eq. (30), strictly speaking, breaks the electromagnetic gauge invariance. The violating terms are, however, of higher orders than we consider. The modified VVCS amplitudes can be found in Appendix B.

The N3LO EFT prediction for the deuteron charge radius, given in Eq. (15), does not change with Eq. (30), as long as we make sure that the parametrisation of the isoscalar nucleon FF agrees with our choice of $r_0^2$. We chose the nucleon FF parametrisations from Borah et al. [35], since their slopes are constrained by the nucleon radii used by us: the proton charge radius from $\mu$H spectroscopy given in Eq. (1a), and the neutron charge radius [36, 37]:

31 $$\begin{aligned} r_n^2=-0.1161(22)\text { fm}^2. \end{aligned}$$

The elastic 2$\upgamma$-exchange correction resulting from inserting the full nucleon FFs can be calculated using Eq. (11) with the re-summed deuteron FFs given in Ref. [21, Eqs. (77) and (78)], and it differs only marginally from the result in Eq. (18) (specifically, by about $-0.001$ meV); we neglect this difference.

The inelastic part changes more significantly. Introducing the nucleon FFs results in the following modifications to the LO and NLO contributions to $E_{2S}^{\textrm{inel},L}$ in Table 1, using the nucleon FF parametrisation of Ref. [35]:

32 $$\begin{aligned} E^{(-3)}\rightarrow E^{(-3)}_\textrm{FF}&=-0.9156~\text {meV}, \nonumber \\ E^{(-2)}\rightarrow E^{(-2)}_\textrm{FF}&= 0.0125~\text {meV}, \end{aligned}$$

which at the same time absorbs both $E ^{(-1)}_{r_N^2}$ and $E ^{(0)}_{r_N^2}$. Using a different nucleon FF parametrisation [38] results in:

33 $$\begin{aligned} E ^{(-3)}\rightarrow E ^{(-3)}_\textrm{FF}&=-0.9151~\text {meV}, \nonumber \\ E^{(-2)}\rightarrow E^{(-2)}_\textrm{FF}&= 0.0125~\text {meV}. \end{aligned}$$

This amounts to a correction of $E _{2S}^\mathrm {hadr,\ FF}=-0.0129$ meV with the nucleon FFs from Ref. [35]; the parametrisation of Ref. [38] gives $E _{2S}^\mathrm {hadr,\ FF}=-0.0121$ meV. In the following, we will adopt

34 $$\begin{aligned} E _{2S}^\mathrm {hadr,\ FF} = -0.013(1)~\textrm{meV}. \end{aligned}$$

This effect is within our N3LO uncertainty estimate; one can also notice that it is significantly larger than a similar difference obtained in a $\chi$ET calculation replacing linearised (expanded in $Q^2$) nucleon FFs by a realistic parametrisation [29]. At the same time, the difference due to the different nucleon FF parametrisations is negligibly small. The replacement of the charge operator by the nucleon FFs in the contributions to $E_{2S}^{\textrm{inel},L}$ beyond NLO would also give a negligible effect on the total result.

**Table 5:** Single-nucleon subtraction-function contributions from 2$\upgamma$ exchange between muon and proton ($\mu$H) or neutron ($\mu n$), respectively. The last column gives the $E _{2S}^\mathrm {hadr,\ subt}$ contribution to $\mu$D, obtained by rescaling the muon-nucleon values. Values are in meV

	$\mu \text {H}$	$\mu n$	$\mu \text {D}$
$\chi$PT [39, 40]	0.0035(26)	0.0043(25)	0.0091(60)
data-driven	0.0023(13) [41]	0.0043(20) [41]	0.0078(37)

Coming to the other effect we consider here, that of the nucleon polarizabilities, it consists of two parts, the inelastic and the subtraction hadronic corrections. The first one of the two can be calculated from a dispersive relation, using the empirical deuteron structure functions, at energies starting from the pion production threshold, as done in Ref. [24]:

35 $$\begin{aligned} E _{2S}^\textrm{hadr, inel}=-0.028(2)~\textrm{meV}. \end{aligned}$$

See also Ref. [42] for a similar evaluation. Another method to calculate it, similar to inserting the nucleon FFs in the consideration above, is to apply a rescaling procedure to the inelastic 2$\upgamma$-exchange effect in $\mu$H and the analogous inelastic 2$\upgamma$-exchange effect between a muon and a neutron ($\mu$n), adding them together and correcting for the different atomic wave functions, rescaling the sum by the factor $[\phi _{2S}^{\mu \textrm{D}}(0)/\phi _{2S}^{\mu \textrm{H}}(0)]^2$, as done in Ref. [5] and references therein. Using the single-nucleon values from Ref. [41], we obtain a value of $-0.030(2)$ meV, where we added uncertainties linearly to be conservative. This perfectly agrees with the dispersive evaluation given by Eq. (35), which is an indication that the rescaling procedure works well in this setting.

The second part of the single-nucleon polarizability effect, the subtraction contribution, cannot be directly related to empirical data. It has to be either modelled or predicted from baryon chiral perturbation theory ($\chi$PT). With the rescaling procedure described above, and the covariant $\chi$PT results for the proton VVCS subtraction function [40] and its neutron counterpart, we obtain for the subtraction-function contribution to $\mu$D^1^:

36 $$\begin{aligned} E _{2S}^\mathrm {hadr,\ subt}=0.009(6)~\textrm{meV}, \end{aligned}$$

which agrees well with the value adopted in Ref. [5]: 0.0098(98) meV. As one can see from Table 5, our predictions agree with the dispersive estimates from Ref. [41]. It is also instructive to compare our result for the proton subtraction contribution, 0.0035(26) meV, to predictions in the framework of heavy-baryon $\chi$PT: 0.0042(10) meV [43] and 0.0029(12) meV [44].

The above considerations take into account the most significant higher-order nucleon structure corrections that start to appear at N4LO in the EFT expansion. One can notice that each one of the corrections, $E _{2S}^\mathrm {hadr,\ FF}=-0.013(1)$ meV from Eq. (34), and the nucleon polarizability corrections, $E _{2S}^\mathrm {hadr,\ subt}+E _{2S}^\mathrm {hadr,\ inel}=-0.019(6)$ meV from Eqs. (35) and (36), is separately smaller or of the size of the estimated N3LO uncertainty of the inelastic contribution, 0.016 meV, Eq. (26). Their total, however,

37 $$\begin{aligned} E _{2S}^\textrm{hadr}= & {} E _{2S}^\mathrm {hadr,\ FF} + E _{2S}^\mathrm {hadr,\ subt} + E _{2S}^\mathrm {hadr,\ inel} \nonumber \\= & {} - 0.032(6)~\textrm{meV}, \end{aligned}$$

is about twice as large as that uncertainty. Nevertheless, we expect the higher-order nuclear effects, as well as the relativistic corrections, to be much smaller, and we expect the remaining higher-order effects to be within our N3LO uncertainty estimate. Erring on the side of caution, we refrain from going as far as performing an N4LO adjustment of the uncertainty.

**Fig. 3:**
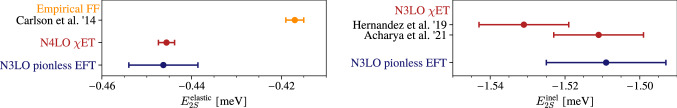
Comparison of predictions for the elastic and inelastic contributions to the 2$\upgamma$ exchange in $\mu$D. Values are the same as in Tables 2 and 4

### Summary of results

We conclude this section by summarizing our EFT predictions of the nuclear-structure effects on the 2*S* level in $\mu$D from the forward 2$\upgamma$ exchange, and including the accompanying electronic VP contributions. At N3LO, we derived the dominant 2$\upgamma$-exchange effects coming from the elastic deuteron charge FF $G_C$ and the non-pole part of the deuteron VVCS amplitude:

38a $$\begin{aligned} E _{2S}^\textrm{elastic}= & {} -0.446(8) \text { meV}, \end{aligned}$$

38b $$\begin{aligned} E _{2S}^{\textrm{inel}}= & {} - 1.509(16) \ \text {meV}, \end{aligned}$$

see Sects. 3.1 and 3.2 for details. The uncertainties have been quantified through the Bayesian error estimate described in Appendix A. As mentioned above, the value of $E_{2S}^\textrm{inel}$ contains the transverse contribution.

In Fig. 3, our EFT predictions are compared to data-driven and $\chi$ET results. The disagreement with Carlson et al. [24] for $E _{2S}^\textrm{elastic}$ is due to the deuteron charge FF parametrisation from Ref. [28]. As one can see from Table 2, our prediction is in good agreement with the data-driven approach if the Sick & Trautmann parametrisation [9] is used instead.

Beyond N3LO, we also take into account the single-nucleon effects discussed in Sect. 3.3. They can be split into the nucleon-polarizability contribution, the single-nucleon subtraction-function contribution, and the insertion of the nucleon FFs in the nucleon charge operator vertex of EFT. In total, they amount to:

39 $$\begin{aligned} E _{2S}^\textrm{hadr} =- 0.032(6)~\textrm{meV}. \end{aligned}$$

On top of the above forward 2$\upgamma$-exchange effects,

40 $$\begin{aligned} E _{2S}^\textrm{fwd}=E_{2S}^\textrm{elastic}+E_{2S}^\textrm{inel}+E_{2S}^\textrm{hadr} =-1.987(20)~\textrm{meV}, \end{aligned}$$

there are the electronic VP corrections to the 2$\upgamma$-exchange, described in Appendix C:

41 $$\begin{aligned} E _{2S}^\textrm{eVP} =-0.027\text { meV} \end{aligned}$$

(their uncertainty also being negligibly small). In total this adds up to:

42 $$\begin{aligned} E _{2S}^{\text {fwd+eVP}} =E _{2S}^\textrm{fwd} + E _{2S}^\textrm{eVP}= -2.014(20)\text { meV}\, . \end{aligned}$$

In Sect. 5, we will discuss all the relevant deuteron-structure effects, including also the Coulomb distortion from the off-forward 2$\upgamma$ exchange [5] and the $3\gamma$-exchange effect [7].

## Hydrogen–deuterium isotope shift

In this section, we will use the isotope shift between 1*S* and 2*S* states in H and D:

43 $$\begin{aligned} E _\text {iso}=h \, f_\text {iso}=E_{2S{-}1S}^\text {D}-E_{2S{-}1S}^\text {H}\,, \end{aligned}$$

where *h* is the Planck constant, to get a prediction for the deuteron charge radius, cf. Eq. (2), and, in turn, determine the LEC $l_1^{C0_S}$ as given by Eq. (17). The empirically measured value of the isotope shift is very precise [45],

44 $$\begin{aligned} f_\textrm{iso}=670\,994\,334.605(15)\,\textrm{kHz}\,. \end{aligned}$$

To extract from it $r_d$ and $l_1^{C0_S}$, we will update the theoretical prediction for the isotope shift. Our notation generally follows the work of Jentschura et al. [45]. It is, along with most of the features of the consideration in this section, such as a list of all contributions relevant to the isotope shift, presented in Appendix D. Here, we focus on our EFT result for the 2$\upgamma$-exchange correction to the *S*-levels in D. The pertinent calculation proceeds analogously to Sect. 3, where the 2$\upgamma$ exchange in $\mu$D is evaluated, hence its details are largely omitted.

### 2$\upgamma$ exchange in deuterium

The longitudinal part of the inelastic contribution to the $2S{-}1S$ shift in D is:

45a $$\begin{aligned} \nu _{9,L}^\textrm{D}= & {} \left[ 16.612 -0.769\, l_1^{C0_S}\right] \textrm{kHz} \end{aligned}$$

45b $$\begin{aligned}= & {} \left[ 9.929+6.825-0.062-0.078\right] \ \textrm{kHz} \nonumber \\= & {} 16.613(191)\ \textrm{kHz}. \end{aligned}$$

In the second line, we show our numerical order-by-order result with the LEC $l_1^{C0_S}$ determined in the following Sect. 4.2. Note that all forward 2$\upgamma$-exchange contributions scale through the atomic wave function at the origin as . Thus, to deduce the shift of the $n^\textrm{th}$ *S*-level in D, one simply has to multiply the isotope shift value by . The uncertainty of our result is obtained in a simplified way by multiplying the total by $(\gamma /m_\pi )^4$. This is justified by the smallness of the N2LO and N3LO contributions (with the NLO contribution given by $(Z-1)$ times the LO result plus a small correction, cf. Table 1 for the case of $\mu$D).

The transverse 2$\upgamma$-exchange contribution appears to be relatively more important in D than in $\mu$D:

46 $$\begin{aligned} \Delta \nu _{9,T}^\textrm{D}&= 1.859(65)\ \textrm{kHz}. \end{aligned}$$

The uncertainty is obtained here by multiplying the total with $(\gamma /m_\pi )^3$, where the usual NLO factor of $(\gamma /m_\pi )^2$ is multiplied with another $\gamma /m_\pi$ to take into account that the transverse amplitude is well reproduced already at NLO [21, Sec. V]. The full N3LO EFT prediction for the inelastic contribution to the forward 2$\upgamma$ exchange is then given by

47 $$\begin{aligned} \Delta \nu _{9,L+T}^\textrm{D} = 18.472(202)\, \textrm{kHz}. \end{aligned}$$

The hadronic contributions to the shift of levels in D are as follows. Inserting the nucleon FFs as in Eq. (30) leads to a negligible shift:

48 $$\begin{aligned} \Delta \nu _{9, \mathrm {\ hadr.\ FF}}^\textrm{D}=0.014(1)\ \textrm{kHz}. \end{aligned}$$

The inelastic part, calculated in the same way as done for $\mu$D [24], gives [46]

49 $$\begin{aligned} \Delta \nu _{9\mathrm {,\ hadr.\ inel}}^\textrm{D}=0.148(11)\ \textrm{kHz}. \end{aligned}$$

This is in perfect agreement with the result from rescaling the single-nucleon values obtained in Ref. [41]: $\Delta \nu _{9\mathrm {,\ hadr.\ inel}}^\textrm{D}=0.145(12)$ kHz. The subtraction part is calculated by us in the same way as done for $\mu$D by rescaling the single-nucleon values from $\chi$PT:

50 $$\begin{aligned} \Delta \nu _{9\mathrm {,\ hadr.\ subt}}^\textrm{D}= -0.069(29)\ \textrm{kHz}. \end{aligned}$$

The subtraction function contributions found in Ref. [41] tend to be smaller, cf. Table 6.

**Table 6:** Single-nucleon subtraction-function contributions from 2$\upgamma$ exchange between electron and proton (H) or neutron (*en*), respectively. The last column gives the $\Delta \nu _{9, \mathrm {\ hadr.\ subt}}$ contribution to D, obtained by rescaling the electron–nucleon values. Values are in kHz

	$\text {H}$	*en*	$\text {D}$
$\chi$PT [39, 40]	$-0.032(15)$	$-0.037(14)$	$-0.069(29)$
data-driven	$-0.016(4)$ [41]	$-0.025(9)$ [41]	$-0.041(13)$

The off-forward 2$\upgamma$-exchange correction, known as the Coulomb distortion, can be estimated by rescaling the results for $\mu$D presented in Ref. [47]. For the $2S{-}1S$ shift in D, we obtain a very small result of $\simeq -0.034$ kHz. Adding all contributions together, we find

51 $$\begin{aligned} \Delta \nu _{9}^\textrm{D} = \left[ 18.530 - 0.769\,l_1^{C0_S} \right] ~\textrm{kHz} = 18.531(204)~\textrm{kHz} . \end{aligned}$$

This has to be compared to $\Delta \nu _{9}^\textrm{D} = 18.70(7)~\textrm{kHz}$ used in Ref. [48] and based on Ref. [49].

The N3LO EFT prediction for the elastic contribution to the $2S{-}1S$ shift in D is

52a $$\begin{aligned} \Delta \nu _{(b)}^\textrm{D}= & {} \left[ 0.625+1.607\,l_1^{C0_S}\right] \textrm{kHz} \end{aligned}$$

52b $$\begin{aligned}= & {} [0.292+0.221+0.087+0.023]~\textrm{kHz}\nonumber \\= & {} 0.622(7)\ \textrm{kHz}, \end{aligned}$$

where the uncertainty is estimated as above for $\Delta \nu _{9,L}^\textrm{D}$. This is slightly bigger than the pure Friar-radius contribution appearing in Ref. [48], which gives $\Delta \nu _{(b)}^\textrm{D}=0.507$ kHz.

Adding all 2$\upgamma$-exchange corrections to the $2S{-}1S$ transition in D together, we find

53 $$\begin{aligned} \Delta \nu _{9+(b)}^\textrm{D}&= \left[ 19.155+0.838\,l_1^{C0_S}\right] ~\textrm{kHz}\nonumber \\&= 19.153(204)\,\, \textrm{kHz} . \end{aligned}$$

One can see that the elastic and inelastic contributions proportional to $l_1^{C0_S}$ partially cancel each other, making the total slightly less sensitive to the value of the N3LO contact term, similarly to what happens in $\mu$D. In any case, the effect of it (assuming the maximal magnitude of $l_1^{C0_S}\simeq 10^{-2}$) is at most $0.01\,\textrm{kHz}$, which is far smaller than the total uncertainty of the isotope shift. Therefore, the contribution of $l_1^{C0_S}$ to the 2$\upgamma$ exchange in the isotope shift can be safely neglected (at the current level of precision), and the deuteron charge radius extracted from the isotope shift is a good quantity to determine $l_1^{C0_S}$.

### 2$\upgamma$ exchange in isotope shift and determination of low-energy constant $l_1^{C0_S}$

For the isotope shift, we also need the 2$\upgamma$-exchange correction to the $2S{-}1S$ transition in H. For the elastic contribution, we use the results from Ref. [41]:

54 $$\begin{aligned} \Delta \nu _{(b)}^\textrm{H} = 0.035(6)~\textrm{kHz}, \end{aligned}$$

which is in perfect agreement with the Friar-radius contribution $\Delta \nu _{(b)}^\textrm{H} =0.035 ~\textrm{kHz}$ appearing in Ref. [48]. For the inelastic contribution, it is important that $\Delta \nu _{9}^\textrm{H}$ is consistent with the single-proton contributions entering D through $\Delta \nu _{9\mathrm {,\ hadr.\ inel}}$ and $\Delta \nu _{9\mathrm {,\ hadr.\ subt}}$. Therefore, we will use the subtraction-function contribution predicted by $\chi$PT, see Table 6, and the inelastic contributions from Ref. [41]:

55 $$\begin{aligned} \Delta \nu _{9}^\textrm{H} = \left[ -0.032(15)+0.073(5)\right] ~\textrm{kHz} = 0.041(16)\,\textrm{kHz}.\nonumber \\ \end{aligned}$$

This compares to $\Delta \nu _{9}^\textrm{H} = 0.061(11)~\textrm{kHz}$ used in Ref. [48] and based on Ref. [50]. Using instead the subtraction-function contribution from Ref. [41], we would find:

56 $$\begin{aligned} \Delta \nu _{9}^\textrm{H}=0.057(6)\,\textrm{kHz}. \end{aligned}$$

In total, the 2$\upgamma$-exchange correction to the $2S{-}1S$ transition in H amounts to

57 $$\begin{aligned} \Delta \nu _{9+(b)}^\textrm{H} = 0.076(17)\,\textrm{kHz}. \end{aligned}$$

The combined results for the isotope shift are given in Eqs. (D19) and (D23) of Appendix D.

In the appendix, we give a full updated list of all contributions entering the isotope shift, together with a comparison to the values used in Ref. [45]. Besides theoretical updates, e.g., of the VP and recoil contributions, we discuss the impact of refined values for the electron, proton and deuteron masses, and the role of the Rydberg constant. Our final result for the theoretical prediction of the $2S{-}1S$ deuterium–hydrogen isotope shift reads:

58 $$\begin{aligned} f_\textrm{iso}&=\Bigg [671\,000\,534.811(924)+ 0.838 \,l_1^{C0_S}\nonumber \\&\quad -1369.346\, \left( \frac{r_d}{\textrm{fm}}\right) ^2\Bigg ]~ \textrm{kHz}. \end{aligned}$$

Note that, in the calculation of the 2$\upgamma$-exchange corrections, we used the value of the proton charge radius $r_p(\mu \text {H})$ published by the CREMA Collaboration, Eq. (1a). This value is consistent with the nucleon FF parametrisations from Ref. [35], used in Sect. 3.3 to estimate the single-nucleon effects beyond N3LO in EFT. The proton finite-size corrections to the isotope shift use instead a refined value [51], extracted from the Lamb shift measurement of the CREMA Collaboration [1, 2] accounting for the recent updates of the $\mu$H theory [52–55]:

59 $$\begin{aligned} r_p(\mu \text {H})=0.84099(36)\,\textrm{fm}. \end{aligned}$$

The effect of the updated $r_p$ value on the 2$\upgamma$-exchange corrections would be negligibly small compared to the estimated theoretical uncertainties of the latter.

The LEC $l_1^{C0_S}$ is small (again, a reasonable estimate of its maximal magnitude being $\simeq 10^{-2}$). It is therefore justified to use the N3LO EFT prediction for the deuteron radius, given in Eq. (15), as an exact relation to express $l_1^{C0_S}$ in Eq. (58) through $r_d$. We can then extract $r_d$ by comparing our theory prediction and the experimental value for the isotope shift (44):

60 $$ \begin{aligned} r_d(\mu \text {H }  \&  \text { iso})=2.12788(16)\ \text {fm}, \end{aligned}$$

where the error is completely dominated by the theory. Our result for $r_d$ is in perfect agreement with the previous extraction in Eq. (2). Setting $l_1^{C0_S}=0$ in Eq. (58) leads to the same result, which proves that the error generated by applying Eq. (15) as an exact relation can indeed be safely neglected. A comparison and consistency check of state-of-the-art deuteron charge radius extractions from $\mu$D, D and the H–D isotope shift can be found in Sect. 6.1. From Eq. (15), we then find:

61 $$\begin{aligned} l_1^{C0_S}=-1.80(4)(36)(12)\times 10^{-3}, \end{aligned}$$

where the uncertainties in the brackets stem from our extracted value of the deuteron radius, the uncertainty of $Z=1.6893(30)$ [56], and the isoscalar nucleon charge radius $r_0=0.5586(10)$ fm, respectively.

## Muonic deuterium Lamb shift

In this section, we will extract an empirical value for the 2$\upgamma$-exchange effects in the $\mu$D Lamb shift from the high-precision Lamb shift measurement by the CREMA Collaboration [3] and the deuteron radius determined from the H–D isotope shift. The empirical value will be compared to our EFT prediction. A theory compilation for the $\mu$D spectrum, including a review of recent theoretical predictions for the 2$\upgamma$-exchange effects, can be found in Ref. [5]. At the end of this section, we will present an updated theory prediction of the $\mu$D Lamb shift, based on our EFT prediction for the 2$\upgamma$ exchange, taking into account all recent theory improvements since the publication of Ref. [5].

### Empirical 2$\upgamma$ exchange

The theory prediction for the $\mu$D Lamb shift reads [5, Eq. (18)]:

62 $$\begin{aligned} E_{2P-2S}= & {} \Bigg [228.77356(75)+0.00310(60) \nonumber \\{} & {} -6.11025(28) \,\left( \frac{r_d}{\textrm{fm}}\right) ^2-E _{2S}^{2\gamma }\Bigg ]\textrm{meV}. \end{aligned}$$

Here, the first term is deuteron-radius independent, the next two terms are deuteron-radius dependent, and the last term contains deuteron-structure effects from 2$\upgamma$ exchange. Note that the prefactor in front of the radius-dependent finite-size term also contains radiative corrections, such as the electronic VP corrections partially discussed in Appendix C, see Ref. [5] for details.^2^ The empirical value measured by the CREMA collaboration is:

63 $$\begin{aligned} E_{2P-2S}=202.8785(31)_\textrm{stat}(14)_\textrm{syst}\, \textrm{meV}. \end{aligned}$$

With the theory prediction for the Lamb shift in Eq. (62), the empirical value in Eq. (63), and $r_d(\mu \text {H }  \&  \text { iso})$ from Eq. (2), one obtains an empirical value for the 2$\upgamma$-exchange effects in the $\mu$D Lamb shift [3]:

64 $$\begin{aligned} E _{2S}^{2\gamma }(\mathrm {emp.})=-1.7638(68)\,\textrm{meV}\,. \end{aligned}$$

In the following, we update this value based on the improved hadronic VP [55] and electronic light-by-light scattering contributions [57], as well as $r_d(\mu \text {H }  \&  \text { iso})$ from Eq. (60).

For the effect of LO and NLO hadronic VP [55], combined with the mixed electronic and muonic VP, as well as the electronic VP loop in the SE correction [52], we use ($2P-2S$): $11.64(32)\,\upmu \textrm{eV}$. This reduces the uncertainty of the old value $11.12(71)\,\upmu \textrm{eV}$ (sum of # #12, 13, 14, 30, 31 in Ref. [5, Table 1]), thereby improving the uncertainty of the deuteron-radius independent term by a factor 2. In addition, we include the inelastic $3\gamma$-exchange, calculated for the first time in Ref. [7]. Compared to Eq. (62), the elastic $3\gamma$-exchange contribution (# #r3, r3$^\prime$ in [5, Table 2]) has been removed from the radius-dependent term, so that the sum of elastic and inelastic $3\gamma$-exchange ($2P-2S$): $2.19(88)(27)\,\upmu$eV [7], is now listed as an individual term. The updated theory prediction for the Lamb shift in $\mu$D then reads [51]:

65 $$\begin{aligned} E_{2P-2S}= & {} \left[ 228.77408(38)-6.10801(28) \,\left( \frac{r_d}{\textrm{fm}}\right) ^2\right. \nonumber \\{} & {} \left. -E _{2S}^{2\gamma }+0.00219(92)\right] \textrm{meV}. \end{aligned}$$

Inserting the deuteron charge radius determined from the H–D isotope shift, Eq. (60), and comparing to the CREMA measurement, Eq. (63), we refine the empirical 2$\upgamma$-exchange effect:

66 $$\begin{aligned} E _{2S}^{2\gamma }(\text {emp.})=-1.7585(56)\,\textrm{meV}. \end{aligned}$$

### Comparison of theoretical predictions for 2$\upgamma$ exchange

**Table 7:** Comparison of prediction for the 2$\upgamma$-exchange effects in the $\mu$D Lamb shift

	$$E _{2S}^{2\gamma }$$ [meV]
Theory prediction	
Krauth et al. [5]	$-1.7096(200)$
Kalinowski [6, Eq. (6) + (19)]	$-1.740(21)$
EFT N3LO	$-1.752(20)$
Empirical ($\mu$H + iso)	
Pohl et al. [3]	$-1.7638(68)$
This work	$-1.7585(56)$

In Sect. 3.4, we summarized our EFT results for the deuteron-structure effects in the $\mu$D Lamb shift originating from the forward 2$\upgamma$ exchange, including the accompanied electronic VP contributions, and compared to other theory predictions. Our final result is given in Eq. (42). For a meaningful comparison to the empirical value for the 2$\upgamma$-exchange effect, Eq. (66), we need to add effects from off-forward 2$\upgamma$ exchange (the Coulomb distortions). Formally of a subleading $O(\alpha ^6\ln \alpha )$, they are, however, numerically important. We use the recommended value from the theory compilation in Ref. [5]:

67 $$\begin{aligned} E _{2S}^\textrm{Coulomb}=0.2625(15)\,\text {meV}, \end{aligned}$$

derived from modern deuteron potentials ($\chi$ET potential and AV18 model [58]). This value should be consistent with the EFT framework, since the deuteron electric dipole polarizability from EFT [21] is in agreement with predictions from the applied deuteron potentials [59]. Combining Eqs. (42) and (67), our final result for the 2$\upgamma$-exchange structure effects on the 2*S*-level in $\mu$D reads:

68 $$\begin{aligned} E _{2S}^{2\gamma } = -1.752(20)\text { meV}, \end{aligned}$$

which is larger than the value accounted for in Ref. [5, Eq. (17)], but agrees with Ref. [6] within errors, cf. Table 7. It is also in agreement with the empirical value, Eq. (66), but more than a factor 3 less precise. Our new theory compilation will be used in Sect. 6.1 to extract $r_d(\mu \text {D})$ from the experimental value for $E_{2P-2S}$.

**Fig. 4:**
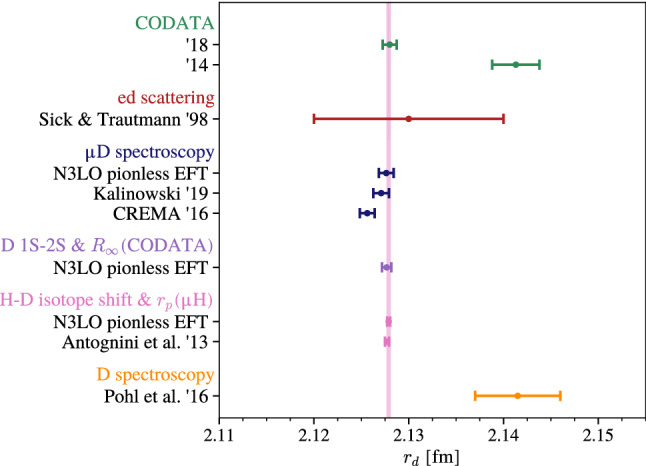
Comparison of deuteron charge radius determinations from fits to electron–deuteron scattering data, ordinary and muonic-deuterium spectroscopy, and the $2S{-}1S$ hydrogen–deuterium isotope shift combined with the proton radius from muonic hydrogen

## Charge radius extractions

### Deuteron charge radius

This section compares three independent extractions of the deuteron charge radius: from the spectroscopy of the $\mu$D Lamb shift, the $2S{-}1S$ transition in D and the $2S{-}1S$ H–D isotope shift, respectively. With the experimental value for the $\mu$D Lamb shift in Eq. (63), the theoretical prediction in Eq. (65), and our result for the 2$\upgamma$-exchange effects, Eq. (68), we can extract the deuteron charge radius from $\mu$D spectroscopy:

69 $$\begin{aligned} r_d(\mu \text {D}) =2.12763 (13)_\text {exp}(77)_\text {theory}= 2.12763(78)~\text {fm},\nonumber \\ \end{aligned}$$

where the uncertainty budget remained the same as in the original extraction from Ref. [3], see Eq. (1b). In addition, we consider the extraction from the measured $2S{-}1S$ transition in D [60]:

70 $$\begin{aligned} f^\textrm{D}_{2S{-}1S}=2\,466\,732\,407\,522.88(91)\,\textrm{kHz}, \end{aligned}$$

and the theory prediction in Eq. (F2), which leads to:

71 $$\begin{aligned} r_d(\text {D}, 2S{-}1S)=2.12767(49)\,\textrm{fm}. \end{aligned}$$

Note that the entering Rydberg constant, $R_\infty$ in Eq. (E4), is strongly driven by $r_p(\mu \text {H})$. The third extraction from the H–D isotope shift and $r_p(\mu \text {H})$ has been presented in Sect. 4.2:

$$ \begin{aligned} r_d(\mu \text {H }  \&  \text { iso})=2.12788(16)\ \text {fm}. \end{aligned}$$

All results are shown in Fig. 4, together will older extractions, results from electron–deuteron scattering and the CODATA recommended values. We can see that the spectroscopy of ordinary and muonic hydrogen isotopes, after the recent theory updates, cf. Ref. [6], gives consistent results for the deuteron charge radius.

### Proton charge radius

Analogously to the calculation of $r_d(\mu \text {H }  \&  \text { iso})$, we can use the isotope shift and $r_d(\mu \text {D})$ to extract the proton charge radius:

72 $$ \begin{aligned} r_p(\mu \text {D }  \&  \text { iso})=0.8404(20)\,\textrm{fm}. \end{aligned}$$

While a previous extraction along these lines, $r_p(\mu \text {D }  \&  \text { iso})=0.8356(20)\,\textrm{fm}$ [3], had been in tension with $r_p(\mu \text {H})$, the result presented here based on the state-of-the-art theory predictions agrees. This, again, nicely shows the consistency between the spectroscopic analyses of ordinary and muonic hydrogen isotopes.

### Proton–deuteron squared charge radii difference

Assuming $m_e \ll M_p \sim M_d$, we can find an approximation for the nuclear-size correction to the H–D isotope shift, Eq. (D22), which is related to the often quoted difference of squared proton and deuteron charge radii. The best such approximation turns out to be

73 $$\begin{aligned} \Delta f_\textrm{iv} \approx -\frac{7}{24\pi }\frac{\alpha ^4 m_e^3 c^4}{\hbar ^3 (1+m_e/M_p)}\left[ r_d^2-r_p^2\right] . \end{aligned}$$

We give here the charge radius difference exactly, based on Eqs. (59) and (60), and use the relation in Eq. (73) only to estimate the uncertainty, which is dominated by the theory of the isotope shift:

74 $$\begin{aligned} r_d^2-r_p^2=3.820\,61(31)\,\textrm{fm}^2. \end{aligned}$$

Using Eq. (73) instead, the central value would decrease by about $1.5\,\sigma$: $3.820\,13\left( {}^{+78}_{-31}\right) \,\textrm{fm}^2$. These results are in good agreement with the difference between charge radii extracted from the Lamb shift in muonic hydrogen isotopes:

75 $$\begin{aligned} r_d^2(\mu \textrm{D})-r_p^2(\mu \textrm{H})=3.819\,55(337)\,\textrm{fm}^2. \end{aligned}$$

From Eq. (74), we can see that the larger CODATA ’14 recommended value for the proton charge radius, $r_p=0.8751(61)\,\textrm{fm}$ [10], would impose a larger value for the deuteron radius inconsistent with the $\mu$D Lamb shift.

## Conclusion and outlook

In this work, we calculated the 2$\upgamma$-exchange corrections to the *S*-levels in ordinary and muonic deuterium in the EFT framework. The calculation was performed at N3LO, with the only unknown LEC $l_1^{C0_S}$ appearing at this order extracted using the H–D isotope shift, where the correlation between that LEC and the 2$\upgamma$-exchange correction is negligible. In addition, we evaluated the contribution of the nucleon structure, i.e., the effect of the nucleon polarizability and of the shape of the nucleon FFs, which are the most important single-nucleon effects beyond N3LO. We also included the accompanying electronic vacuum polarization contributions.

Our predictions for the elastic contribution to the 2$\upgamma$ exchange in $\mu$D from EFT at N3LO and $\chi$ET at N4LO appear to be several standard deviations larger than the evaluations [24, 29] based on the deuteron charge FF parametrisation of Ref. [28], cf. Fig. 3 and Table 2. This suggests that the latter parametrisation does not adequately describe the behaviour of the deuteron charge FF at low virtualities. The correlation between the Friar radius $r_{\textrm{F}d}$ and the deuteron charge radius $r_d$ in EFT, cf. Fig. 2, through the LEC $l_1^{C0_S}$ could serve as a diagnostic criterion for a realistic parametrisation of the deuteron charge FF. We also point out that the EFT expression for the deuteron charge FF at N3LO [21, Sec. IV] can be used for an analytic one-parameter fit to the electron–deuteron scattering data in the low-$Q^2$ range relevant to the planned DRad experiment [31].

Supplementing the $\mu$D theory [5] with a few missing electronic VP effects [6] and the inelastic $3\gamma$ exchange [7], together with the shift of the elastic contribution found in this work, the past discrepancy between theory and experiment on the size of 2$\upgamma$-exchange effects, see Table 7, is now completely resolved.

The uncertainty of the theoretical result for the $2\gamma$-exchange correction was quantified using Bayesian inference. While our N3LO EFT prediction was not yet able to improve the theoretical precision, the improved understanding of the elastic contribution is of utmost importance. In addition, by calculating the 2$\upgamma$-exchange correction to $\mu$D and D, we were able to perform a few consistency checks. In particular, we showed that extractions of the deuteron charge radius from the $\mu$D Lamb shift, the $2S{-}1S$ transition in D and the $2S{-}1S$ H–D isotope shift, cf. Fig. 4, are now in excellent agreement.

## Figures and Tables

**Fig. 5:**
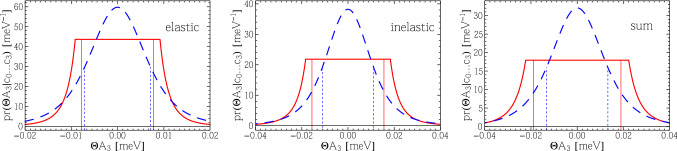
Probability distribution functions for $\Theta (E _{2S}^\textrm{elastic})$ (left), $\Theta (E _{2S}^{\textrm{inel},L})$ (center), and $\Theta (E _{2S}^\textrm{sum})$ (right). Red solid (blue dashed) curves correspond to Set A (Set C) priors. Thin vertical lines indicate the corresponding 68% degree-of-belief intervals

**Fig. 6:**
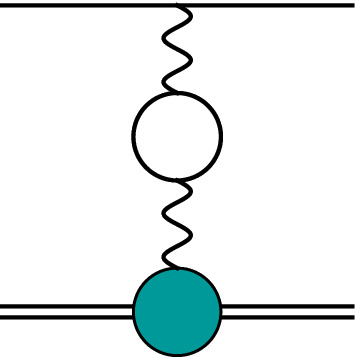
One-photon exchange with vacuum-polarization and finite-size correction

**Fig. 7:**
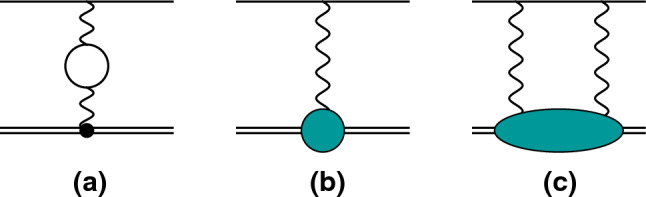
**a** One-photon exchange with vacuum polarization; **b** one-photon exchange with finite-size correction; and **c** elastic and inelastic two-photon exchange

**Fig. 8:**
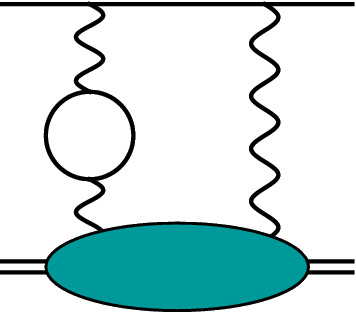
Elastic and inelastic two-photon exchange with vacuum-polarization insertion at $O(\alpha ^6)$

## Data Availability

This manuscript has no associated data or the data will not be deposited. [Authors’ comment: No data is associated with this manuscript. The associated program codes will be available from the authors upon request.]

## Appendix A: Quantification of uncertainty

The uncertainty of an EFT calculation is in many cases, including the present work, dominated by the unknown higher-order terms rather than input parameters. To quantify the uncertainty, we follow the Bayesian approach developed in Refs. [20, 63] and references therein. The results and the details specific to our evaluation are presented in this section.

We start with the EFT expansion of a generic observable *A* in powers of the expansion parameter $\xi$ (which is $\xi =\gamma /m_\pi$ in EFT):

A1 $$\begin{aligned} A = A_0\sum \limits _{n=0}^\infty c_n\xi ^n, \end{aligned}$$

where the parameter $A_0$ sets the scale of *A*, and $c_n$ are the expansion coefficients. The uncertainty of *A* caused by a truncation at $n=k$ is given by the unknown remainder:

A2 $$\begin{aligned} \Theta A_k = A_0\sum \limits _{n=k+1}^\infty c_n\xi ^n. \end{aligned}$$

The typical prior assumption used in EFT calculations is the naturalness of the expansion coefficients, i.e., $c_n$ should be at most *O*(1). One may refine this assumption using the calculated expansion coefficients as done in, e.g., Ref. [64] that assigns (written out here for an N3LO calculation, $k=3$):

A3 $$\begin{aligned} \Theta A_3&= \max \Big \{\xi ^4|\Delta A^\textrm{LO}|, \xi ^3|\Delta A^\textrm{NLO}|,\xi ^2|\Delta A^\textrm{N2LO}|,\nonumber \\&\quad \xi |\Delta A^\textrm{N3LO}|\Big \}\nonumber \\&=A_0\,\xi ^4 \max \limits _{n\le 3} |c_n|, \end{aligned}$$

where $\Delta A^\textrm{LO}$, $\Delta A^\textrm{NLO}$, etc. are the contributions to *A* at the respective order. This also implicitly assumes that $\Theta A_k$ is dominated by its first term, $A_0\, c_{k+1}\xi ^{k+1}$; we will employ this assumption in the following (note that it can be relaxed, in particular, in the Bayesian approach [20, 63]). Looking at the expansion of $E _{2S}$, we note that the expansion of the LSZ factor, $\left[ \Sigma '(E_d)\right] ^{-1}\propto 1 + (Z-1) + 0 + 0 + \cdots$, see Ref. [21, Eq. (49)], results in every term appearing at a given order in the four-point function acquiring a factor of *Z* at the next order, as is also explicitly shown in Table 1. This induces correlations between the coefficients in the expansion. It is therefore natural to slightly modify the expansion for the purpose of quantifying the uncertainty, taking out the known *Z* factor along with the scale factor $A_0$. This amounts to working with the expansion of the (integrals of) the four-point functions, and the simple estimate of Eq. (A3) becomes:

A4 $$\begin{aligned} \Theta A_3&= Z A_0 \,\xi ^4 \max \limits _{n\le 3}|c_n|, \end{aligned}$$

where $A_0$ and $c_n$ are now the normalization and the expansion coefficients of the integrals of the four-point function, which can be deduced from Table 1, respectively, for $E _{2S}^\textrm{elastic}$ and $E _{2S}^{\textrm{inel},L}$.

**Table 8:** Expansion coefficients and prefactors corresponding to the modified EFT expansion

	$A_0$ [meV]	$\qquad c_0$	$c_1$	$c_2$	$c_3$
$E _{2S}^\textrm{elastic}$	$-0.204$	1	0.260	2.311	-1.896
$E _{2S}^{\textrm{inel},L}$	$-0.943$	1	-0.050	-0.379	0.044
$E _{2S}^\textrm{sum}$	$-1.148$	1	0.006	0.100	-0.301

Moving to the Bayesian quantification, we use the prior probability density functions (PDFs) introduced in Ref. [20] as Sets A, B, and C. Each one of the sets of priors consists of two PDFs, $\textrm{pr}(c)$ and $\textrm{pr}(c_n|c)$, where the first is associated with the scale *c* typical of the coefficients $c_n$, while the second describes the probability distribution of $c_n$ given the scale. In the leading-term approximation for the uncertainty, the Bayes’ theorem with the given prior PDFs gives the PDF of the uncertainty at order *k*, given the known $c_0,\dots c_k$:

A5 $$\begin{aligned} \textrm{pr}(\Theta A_k|c_0,\dots c_k) = \frac{\int \limits _0^\infty \textrm{d}c\, \textrm{pr}(c_{k+1}|c)\, \textrm{pr}(c)\prod \nolimits _{m=0}^k \textrm{pr}(c_m|c)}{Z\,A_0\,\xi ^{k+1}\int \limits _0^\infty \textrm{d}c\, \textrm{pr}(c)\prod \nolimits _{m=0}^k \textrm{pr}(c_m|c)} \nonumber \\ \end{aligned}$$

with

A6 $$\begin{aligned} c_{k+1} = \frac{\Theta A_k}{Z\,A_0\,\xi ^{k+1}}, \end{aligned}$$

where we specialize to the case of $E _{2S}$ and the quantities $A_0$ and $c_n$ again pertain to the expansion of the (integrals of) the four-point function (with the *Z* factored out).

The expansion coefficients and prefactors that correspond to the modified EFT expansion of $E _{2S}^\textrm{elastic}$, $E _{2S}^{\textrm{inel},L}$, and their sum $E _{2S}^\textrm{sum} = E _{2S}^\textrm{elastic}+E _{2S}^{\textrm{inel},L}$, are given in Table 8. One can see that the expansion coefficients of the elastic contribution are somewhat larger than one, whereas the inelastic part, as well as the total, have smaller expansion coefficients. There are partial cancellations between the higher-order elastic and inelastic contributions, which, together with the LO total term being about five times larger than its elastic counterpart, suppresses the expansion coefficients of the total 2$\upgamma$-exchange correction. With these coefficients, we start with the estimate of Eq. (A4), which gives:

A7 $$\begin{aligned}{} & {} \Theta \left( \left\{ E _{2S}^\textrm{elastic},E_{2S}^{\textrm{inel},L},E_{2S}^\textrm{sum}\right\} \right) \nonumber \\{} & {} \quad = \{0.009,\,0.018,\,0.022 \}\ \text {meV}. \end{aligned}$$

The uncertainties deduced this way are a priori not Gaussian-distributed quantities (as one sees explicitly from, e.g., the Bayesian PDFs below, cf. Fig. 5), therefore the usual addition of uncertainties in quadrature may not be an adequate way to, e.g., calculate the uncertainty of a sum given the uncertainties of its constituents; we therefore estimate the uncertainty of $E _{2S}^\textrm{sum}$ independently.

Proceeding to the Bayesian estimates, we apply Eq. (A5) with the priors of Ref. [20] to the results of our calculation. The specific parameters that we use for the priors are:

A8 $$\begin{aligned} c_>=100,\ c_<=10^{-3},\ \sigma = 2.0. \end{aligned}$$

The resulting PDFs for $\Theta A_3$ are shown in Fig. 5. Set A and Set B result in PDFs that in each case are practically on top of each other, we therefore show only Set A as a representative, along with Set C.

The Bayesian procedure results in the following $68\%$ degree-of-belief (DOB) intervals:

A9a $$\begin{aligned}&\text {Set A}: \qquad \Theta \left( \left\{ E _{2S}^\textrm{elastic},E_{2S}^{\textrm{inel},L},E_{2S}^\textrm{sum}\right\} \right) \nonumber \\&\quad = \{0.0078,\,0.0156,\,0.0189 \}\ \text {meV}, \end{aligned}$$

A9b $$\begin{aligned}&\text {Set B}: \qquad \Theta \left( \left\{ E _{2S}^\textrm{elastic},E_{2S}^{\textrm{inel},L},E_{2S}^\textrm{sum}\right\} \right) \nonumber \\&\quad = \{0.0077,\,0.0155,\,0.0188 \}\ \text {meV}, \end{aligned}$$

A9c $$\begin{aligned}&\text {Set C}: \qquad \Theta \left( \left\{ E _{2S}^\textrm{elastic},E_{2S}^{\textrm{inel},L},E_{2S}^\textrm{sum}\right\} \right) \nonumber \\&\quad = \{0.0071,\,0.0111,\,0.0133 \}\ \text {meV}. \end{aligned}$$

One can see that the simple estimate of Eq. (A7) gives a more conservative uncertainty than is obtained from the Bayesian framework, which was also the tendency observed in Ref. [20]. It is also evident that the priors Set A and B result in somewhat bigger uncertainties than Set C; the difference is rather small for the elastic contribution, but is of the order of 25% for the inelastic term and the total 2$\upgamma$-exchange correction. Seeing that the current uncertainty estimate uses the leading omitted term approximation, one can expect an about 30% change in the uncertainty once higher-order terms are taken into account. The above difference between Sets A and B, on the one hand, and Set C, on the other hand, is thus not unexpectedly big. One can also note that the uncertainty of the elastic term is probably overestimated, since higher-order terms are unlikely to change that term as much as suggested by the projected uncertainty, given that the value of $r_d^2$ is fixed at N3LO. This is also supported by the agreement between the EFT and $\chi$ET results for the elastic contribution. We take the more conservative results of Sets A and B as our uncertainty estimate for the calculated 2$\upgamma$-exchange contributions.

Finally, one may notice that, in particular, the relative smallness of the higher-order coefficients in Table 8, might mean that the assignment $\xi =1/3$ overestimates the size of the expansion parameter (and thus also the truncation uncertainty). As demonstrated in, e.g., Ref. [20] with $\chi$ET calculations, Bayesian tools could also be applied to quantify the (assigned) value of the expansion parameter. Such a study would ideally involve additional quantities obtained in the same EFT framework, and will be presented elsewhere; in this context, see Ref. [65] that studies the uncertainties arising from a different expansion employed in calculations of 2$\upgamma$-exchange corrections in muonic atoms and ions.

## Appendix B: Deuteron VVCS amplitudes with insertion of nucleon form factors

In this appendix, we provide the expressions for the longitudinal deuteron VVCS amplitude at LO and NLO resulting from the procedure outlined in Sect. 3.3, where one inserts the full nucleon FFs. The expressions for the four-point function (see Ref. [21, Sec. II D] for the definition) with the inserted FFs read:

B1 $$\begin{aligned} {\mathcal {M}}_L^\mathrm {(-3),\ FF}&= \frac{e^2 M^3}{\pi }\frac{Q^2}{\varvec{q}^2} \left\{ \frac{\left[ {\bar{G}}_E^0(Q^2)\right] ^2+\left[ {\bar{G}}_E^1(Q^2)\right] ^2}{\gamma \left[ \varvec{q}^2+4(\gamma +\lambda _d)^2\right] }\right. \nonumber \\&\quad +\frac{\left[ {\bar{G}}_E^0(Q^2)\right] ^2-\left[ {\bar{G}}_E^1(Q^2)\right] ^2}{M|\varvec{q}|\nu }\phi (\nu ,\varvec{q}^2)\nonumber \\&\quad \left. -\frac{4\left[ {\bar{G}}_E^0(Q^2)\right] ^2}{\varvec{q}^2\left( \gamma -\lambda _d\right) }\phi ^2(\nu ,\varvec{q}^2) \right\} \nonumber \\&\quad +(\nu \rightarrow -\nu ), \end{aligned}$$

B2 $$\begin{aligned} {\mathcal {M}}_L^\mathrm {(-2),\ FF}&=\frac{e^2 M^3}{\pi }\frac{Q^2}{\varvec{q}^2} \frac{2(Z-1)}{\gamma }\left[ \bar{G}_E^0(Q^2)\right] ^2\nonumber \\&\quad \times \frac{\phi (\nu ,\varvec{q}^2)\left[ |\varvec{q}|-(\gamma +\lambda _d)\phi (\nu ,\varvec{q}^2) \right] }{\varvec{q}^2(\gamma -\lambda _d)} \nonumber \\&\quad +(\nu \rightarrow -\nu ). \end{aligned}$$

Here, the kinematic functions are [21]:

B3 $$\begin{aligned} \lambda _d\!=\!\sqrt{\gamma ^2-M\nu +\frac{\varvec{q}^2}{4}}, \phi (\nu ,\varvec{q}^2)\!=\!\arctan \frac{\left| \varvec{q}\right| }{2(\gamma +\lambda _d)}, \end{aligned}$$

and the barred nucleon isoscalar and isovector electric FFs are:

B4 $$\begin{aligned} {\bar{G}}_E^{0,1}(Q^2)=\frac{G_E^{0,1}(Q^2)}{\sqrt{1+\frac{Q^2}{4M_p^2}}}. \end{aligned}$$

## Appendix C: Electronic vacuum polarization corrections to finite-size and polarizability contributions

In this appendix, we consider the one-loop electronic VP given by:

C1 $$\begin{aligned} \overline{\Pi }^{(1)}(Q^2)&= \Pi ^{(1)}(Q^2)-\Pi ^{(1)}(0)\nonumber \\&= \frac{\alpha }{3\pi } \left[ 2 \left( 1-\frac{1}{2\tau _e }\right) \right. \nonumber \\&\quad \times \,\left. \left( \sqrt{1+\frac{1}{\tau _e }} {{\,\textrm{arccoth}\,}}\sqrt{1+\frac{1}{\tau _e }} -1\right) +\frac{1}{3}\right] , \end{aligned}$$

with $\tau _e=Q^2/4m_e^2$ and $m_e$ the electron mass, and its well known corrections to the deuteron structure effects.

We start with the corrections to the $O(\alpha ^4)$ deuteron radius term to illustrate the approach, before calculating the corrections to the $O(\alpha ^5)$ 2$\upgamma$-exchange effect, relevant for this paper. The one-loop electronic VP correction to the deuteron charge radius term, see Fig. 6, is described by the following potential:

C2 $$\begin{aligned} \Delta V_\text {VP-FF}(r)= & {} - \int \!\frac{\textrm{d}\varvec{q}}{(2\pi )^3} \, e^{i \varvec{q}\cdot \varvec{r}} \, \frac{4\pi \alpha }{\varvec{q}^2} \, \overline{\Pi }^{(1)}(\varvec{q}^2)\nonumber \\{} & {} \times \left[ G_C(\varvec{q}^2) -1\right] . \end{aligned}$$

Note that this is a contribution to the Breit potential [66, Ch. IX, §83], where the retardation effects can be neglected at this order in $\alpha$, and hence $Q^2$ is replaced by $\varvec{q}^2$. We shall make use of the dispersion relations (DRs) for the VP and the FF:

C3a

C3b

where  denotes the principal-value integration. The one-loop expression for the absorptive part of electronic VP reads:

C4 $$\begin{aligned} {{\,\textrm{Im}\,}}\, \Pi ^{(1)}(t) = -\frac{\alpha }{3}\left( 1+\frac{2m_e^2}{t}\right) \sqrt{1-\frac{4m_e^2}{t} }. \end{aligned}$$

The DR for VP is once-subtracted to ensure the correct normalisation of the electromagnetic field. Similarly to ensure the correct normalisation of the deuteron charge, $G_C(0)=1$, we can use the once-subtracted relation for the charge FF:

C5

In first-order perturbation theory to the unperturbed Coulomb wave functions, one finds for the Lamb shift:

C6

It is clear that the dominant effect comes from the small-*t* region in the first integral, which starts from the threshold of $e^+ e^-$ production. Unfortunately, we cannot simply expand $G_C$ around 0 before integration, since the integral will eventually diverge. Instead we use again the DR for $G_C$ given in Eq. (C3a). We then change the variable $t\rightarrow 4m_e^2 u^2$ and perform the integration over *u*. Afterwards, only integrals over $t'$ remain, which start from the threshold of hadron (e.g., $\pi ^+ \pi ^-$) production $t_0$. Assuming that $2 m_e \ll t_0 \le t'$, we can expand up to $O(4m_e^2/t' )$. Neglecting terms which are suppressed by additional factors of $m_e^2$, we obtain:

C7a $$\begin{aligned} E _{2P-2S}^{(1)\langle \text {VP-FF}\rangle }&=-\frac{1}{6}\, \alpha ^5 m_r^3 A(\kappa ) r_d^2 \end{aligned}$$

C7b $$\begin{aligned}&=-0.0135\, \left[ \frac{r_d}{\text {fm}}\right] ^2 \, \text{ meV }\nonumber \\&\simeq -0.06113(1) \, \text{ meV }, \end{aligned}$$

with the auxiliary function:

C8 $$\begin{aligned} A(\kappa )&= \frac{1}{12 \pi (1-\kappa ^2)^2} \left[ \kappa ^2( 4\kappa ^2 - 7) \right. \nonumber \\&\quad \left. + \frac{\kappa ( 4 \kappa ^4-10 \kappa ^2+9) }{\sqrt{1-\kappa ^2}} \arccos \kappa \right] \nonumber \\&\simeq 0.152309 \end{aligned}$$

at $\kappa =\alpha m_r/2m_e$. Our formula agrees numerically with Ref. [67, Eq. (28)]. In Eq. (C7b), we used the deuteron radius determined through the isotope shift to illustrate the quantitative size of the effect, where the uncertainity is just propagated from the error of the radius in Eq. (60).

A similar subleading correction stems from the interference of one-photon exchange potentials with electronic VP,

C9

and finite-size corrections,

C10 $$\begin{aligned} \Delta V_{\textrm{FF}} (r)&= - \int \!\frac{\textrm{d}\varvec{q}}{(2\pi )^3} \, e^{i \varvec{q}\cdot \varvec{r}} \, \frac{4\pi \alpha }{\varvec{q}^2} \, \left[ G_C(\varvec{q}^2) - 1\right] \nonumber \\&\simeq \frac{4\pi \alpha \,r_d^2}{6} \,\delta (\varvec{r}), \end{aligned}$$

see Fig. 7a, b, respectively. The latter can be approximated with a delta-function potential proportional to the deuteron radius. To calculate this effect at second order in perturbation theory, we need to know the matrix elements of the delta-function and Yukawa-type potentials between the $\mu$D Coulomb wave functions:

C11a

C11b

and the energy levels of the Coulomb potential:

C12 $$\begin{aligned} E_n=-\frac{\alpha }{2 a n^2}, \qquad E_2=-\frac{\alpha }{8a}, \end{aligned}$$

with *n* the principal quantum number. For the discrete spectrum, we obtain:

C13

C14

C15

with the deuteron radius in fm units. For the continuous spectrum, we apply:

C16a

C16b

and

C17 $$\begin{aligned} E_k=\frac{\alpha k^2}{2 a}, \end{aligned}$$

to get:

C18

C19

C20

In total, the interference of the one-photon-exchange potentials in Fig. 7a, b amounts to:

C21 $$\begin{aligned} E ^{(2) \langle \text {VP} \rangle \langle \text {FF} \rangle }_{2S}&=E ^{(2) \mathrm { disc.}\langle \text {VP} \rangle \langle \text {FF} \rangle }_{2S}+E ^{(2) \mathrm { cont.}\langle \text {VP} \rangle \langle \text {FF} \rangle }_{2S} \end{aligned}$$

C22 $$\begin{aligned}&=0.020487\, \left[ \frac{r_d}{\text {fm}}\right] ^2 \, \text{ meV }\nonumber \\&\simeq 0.092763(14)\,\textrm{meV}. \end{aligned}$$

This formula agrees numerically with Ref. [67, Eq. (29)].

Let us now turn to our main interest: the electronic VP corrections to the 2$\upgamma$ exchange. The simplest correction is due to the insertion of the one-loop electronic VP into the 2$\upgamma$-exchange diagram, see Fig. 8. We multiply the integrand in Eq. (9a) with $\left[ 1-\overline{\Pi }^{(1)}(Q^2)\right] ^{-2}$ and obtain for the sum of elastic and inelastic contributions:

C23 $$\begin{aligned} E _{2S}^{(1)\langle 2\gamma \text {-}\textrm{VP}\rangle }&=[-0.0071-0.0136]~\textrm{meV}\nonumber \\&= -0.0207\text { meV}. \end{aligned}$$

In addition, there is a correction to the $\mu$D atomic wave function that can be calculated at second order in perturbation theory from the interference of the one-photon exchange potential with VP insertion, Eq. (C9), and the forward 2$\upgamma$-exchange potential:

C24 $$\begin{aligned} \Delta V_{{2\gamma }} (r)=\frac{E ^\text {fwd}_{nS}}{[\phi _n(0)]^2}\,\delta (\varvec{r}), \end{aligned}$$

see Fig. 7a, c, respectively. Since the latter is a delta-function potential just like our approximated one-photon exchange potential with finite-size correction, Eq. (C10), the calculation of $E ^{(2) \langle \text {VP} \rangle \langle 2\gamma \rangle }_{2S}$ proceeds analogously to the calculation of $E ^{(2) \langle \text {VP} \rangle \langle \text {FF} \rangle }_{2S}$ above. We therefore present here only the results:

C25a $$\begin{aligned} E ^{(2) \mathrm { disc.}\langle \text {VP} \rangle \langle 2\gamma \rangle }_{2S}&= 0.0013624 \, E ^\text {fwd}_{2S}\nonumber \\&\simeq -0.00271\,\textrm{meV}, \end{aligned}$$

C25b $$\begin{aligned} E ^{(2) \mathrm { cont.}\langle \text {VP} \rangle \langle 2\gamma \rangle }_{2S}&= -0.0047358\, E ^\text {fwd}_{2S}\nonumber \\&\simeq 0.00941\,\textrm{meV}, \end{aligned}$$

C25c $$\begin{aligned} E ^{(2) \langle \text {VP} \rangle \langle 2\gamma \rangle }_{2S}&=E ^{(2) \mathrm { disc.}\langle \text {VP} \rangle \langle 2\gamma \rangle }_{2S} \!+\!E ^{(2) \mathrm { cont.}\langle \text {VP} \rangle \langle 2\gamma \rangle }_{2S} \end{aligned}$$

C25d $$\begin{aligned}&=1.4523\,\frac{\alpha }{\pi }\, E ^\text {fwd}_{2S}=0.0033734\, E ^\text {fwd}_{2S}\nonumber \\&=-0.00670(7)\,\textrm{meV}. \end{aligned}$$

The formula agrees numerically with the wave function correction in Refs. [6, Eqs. (17) and (18)] and [54, Table II]. Here we used the full forward 2$\upgamma$-exchange result, $E _{2S}^\text {fwd}=-1.987(20)$ meV, collected in Eq. (40), and propagated its uncertainty into Eq. (C25d).

The sum of electronic VP corrections to the 2$\upgamma$ exchange, amounts to:

C26 $$\begin{aligned} E _{2S}^\textrm{eVP}= & {} E _{2S}^{(1)\langle 2\gamma -\textrm{VP}\rangle }+E^{(2) \langle \text {VP} \rangle \langle 2\gamma \rangle }_{2S}\nonumber \\= & {} -0.0274\text { meV}, \end{aligned}$$

which is about a factor one-and-a-half larger than our error estimate for $E _{2S}^\text {fwd}$. Our result is comparable to the results of Ref. [6, Eq. (19)]: $E _{2S}^\textrm{eVP}=-0.0265(3)$ meV.

## Appendix D: Hydrogen–deuterium isotope shift

In this appendix, we will update the analysis of the H–D isotope shift presented in Ref. [45], which is based on the framework reviewed in the CODATA 2006 report [48]. More recent summaries of the theory of hydrogen-like atoms can be found, e.g., in Refs. [7, 68]. The most relevant physical constants entering the isotope shift calculation are listed below, with their relative uncertainties given in square brackets:

D1 $$\begin{aligned} m_e&=9.109\,383\,7015(28)\times 10^{-31}\;\text {kg}&[3.1\times 10^{-10}],\nonumber \\ \frac{m_e}{M_p}&=5.446\,170\,214\,87 (33)\times 10^{-4}&[6.0\times 10^{-11}],\nonumber \\ \frac{m_e}{M_d}&=2.724\,437\,107\,462 (96)\times 10^{-4}&[3.5\times 10^{-11}],\nonumber \\ \frac{M_d}{M_p}&=1.999\,007\,501\,39 (11)&[5.6\times 10^{-11}],\nonumber \\ \alpha ^{-1}&=137.035\,999\,084 (21)&[1.5\times 10^{-10}],\nonumber \\ R_\infty c&=3.289\,841\,960\,2508 (64)\times 10^{15}\; \text {Hz}&[1.9\times 10^{-12}]. \end{aligned}$$

The values are taken from the latest CODATA and PDG reports [4, 69]. Compared to the CODATA 2006 report [48], the relative uncertainties have improved by a factor 3.5 to 12. The relative uncertainty of $m_e$ has even shrunk by two orders of magnitude. The Rydberg constant, $R_\infty$, listed above is a result of the CODATA 2018 fit of various measured transitions in hydrogen-like atoms. It is, however, largely driven by the Lamb shift in $\mu$H. A similar result is obtained relying solely on the Lamb shift in $\mu$H and the $2S{-}1S$ transition in H, see our determination in Appendix E.

In Ref. [45], the various contributions to the isotope shift are split into four Sets (i)–(iv). The bulk of the isotope shift comes from the respective difference of the Dirac eigenvalues once the reduced mass effects are accounted for, identified in Ref. [45] as Set (i). Based on the newest CODATA 2018 set of physical constants, we evaluate the frequency shift as:

D2 $$\begin{aligned} \Delta f_\textrm{i}&= 671\,004\,071.028(85)~\textrm{kHz}\nonumber \\&\quad [671\,004\,071.29(66)~\textrm{kHz}]. \end{aligned}$$

This is 262 Hz smaller than the old value (given in square brackets) obtained using the CODATA 2006 set of constants, and considerably more precise. As mentioned already, the best values for the Rydberg constant, see Eqs. (D1) and (E4), are determined i.a. from the experimental $2S{-}1S$ transition in H, thus, rely on the same theory input as the $2S{-}1S$ H–D isotope shift. However, since they are largely driven by $r_p(\mu \text {H})$, it is justified to use these values in our analysis. Both central value and uncertainty estimate, following Ref. [45], are the same for $\Delta f_\textrm{i}$ with Eqs. (D1) and (E4), respectively.

We should also mention that since the latest CODATA adjustment, there have been many advances in the experimental determination of the electron–proton mass ratio [70–72] and the proton–deuteron mass ratio [73, 74]. The presently most precise values (relative uncertainty in square brackets),

$$\begin{aligned} \frac{m_e}{M_p}&=5.446\,170\,214\,805 (98)\times 10^{-4}&[1.8\times 10^{-11}],\\ \frac{M_d}{M_p}&=1.999\,007\,501\,243 (31)&[1.6\times 10^{-11}], \end{aligned}$$

are based on an improved theory of rovibrational spin-averaged transitions in the hydrogen molecular ion $\text {HD}^+$ [75]. From this it also follows that:

$$\begin{aligned} \frac{m_e}{M_d}&=2.724\,437\,107\,624 (65)\times 10^{-4}&[2.4\times 10^{-11}]. \end{aligned}$$

Using these values to calculate the effect of the dominant Dirac eigenvalue contribution to the isotope shift, we obtain:

D3 $$\begin{aligned} \Delta f_\textrm{i} =671\,004\,070.972(29)~\textrm{kHz}, \end{aligned}$$

in agreement with Eq. (D2), but almost a factor three more precise. Note that the $5\,\sigma$ variance between the latest determination of the fine structure constant $\alpha$ from a rubidium recoil measurement [76] and the best caesium recoil measurement [77] has no effect on the isotope shift.

The next by size Set (ii) of Ref. [45] includes ten contributions with frequency shifts $\Delta \nu _j$. We checked that we reproduce the numbers for this set from Ref. [45] individually, using the constants and the formalism given in the CODATA 2006 report [48], up to $0.01~\textrm{kHz}$. The new CODATA 2018 constants do not have a significant effect here. However, there have been considerable improvements of the theory, as we show in our updated evaluation below (old values in square brackets):

1. One-loop SE and electronic VP:
  D4 $$\begin{aligned} \Delta \nu _1=-5558.999\,\text {kHz}\qquad [-5558.99\,\text {kHz}]. \end{aligned}$$
2. Two-loop SE, electronic VP, and combined effects:
  D5 $$\begin{aligned} \Delta \nu _2=-0.521(1)\,\text {kHz}\qquad [-0.51\,\text {kHz}]. \end{aligned}$$
  Here, we use updated values for the coefficients [10]:
  D6 $$\begin{aligned} B_{50}(nS)&=-21.554 47(13)&[-21.5561(31)], \end{aligned}$$
  D7 $$\begin{aligned} B_{60}(1S)&=-81.3(0.3)(19.7)&[-95.3(0.3)(33.7)], \end{aligned}$$
  D8 $$\begin{aligned} B_{60}(2S)&=-66.2(0.3)(19.7)&[-80.2(0.3)(33.7)]. \end{aligned}$$
  For the logarithmic coefficient $B_{61}$, we are including previously neglected light-by-light contributions, evaluated in Ref. [78, 79], see Ref. [80]:
  D9 $$\begin{aligned} B_{61}^\text {LbL}(nS)&=-\frac{43}{36}+\frac{709 \pi ^2}{3456}. \end{aligned}$$
  We checked that using instead of $B_{60}(1S)$ the all-order in $({\mathcal {Z}} \alpha )$ coefficient [80],^3^
  D10 $$\begin{aligned} G_{60}(1S)&=-94.5(6.6), \end{aligned}$$
  the result changes marginally, to $-0.520$ kHz. This is within the uncertainty estimate in Eq. (D5). For a discussion of the pure SE contribution to $G_{60}$ see Ref. [81].
3. Three-loop SE, electronic VP, and combined effects:
  D11 $$\begin{aligned} \Delta \nu _3=-0.001\,\text {kHz}\qquad [-0.001\,\text {kHz}]. \end{aligned}$$
  Including $C_{50}$ and $C_{62}$ from Ref. [82, 83], see Ref. [80]:
  D12 $$\begin{aligned} C_{50}(nS)&=-3.3(10.5)&[\pm \, 30], \end{aligned}$$
  D13 $$\begin{aligned} C_{62}(nS)&=-\frac{2}{3}B_{40}(nS)\simeq -0.36,&\end{aligned}$$
  in addition to the leading $C_{40}$ term, has no effect at the precision given above. Note that the four-loop QED contribution has been calculated in Ref. [84], but can be neglected at the present level of precision.
4. Salpeter recoil correction:
  D14 $$\begin{aligned} \Delta \nu _4=1032.65\,\text {kHz}\qquad [1032.65\,\text {kHz}]. \end{aligned}$$
5. Higher-order pure recoil corrections:
  D15 $$\begin{aligned} \Delta \nu _5= & {} (-3.140+0.081)\,\,\text {kHz}\nonumber \\= & {} -3.059(7)\,\text {kHz}\qquad [-3.41(32)\,\,\text {kHz}]. \end{aligned}$$
  In Ref. [45], pure recoil corrections at first order in the electron–nucleus mass ratio and expanded up to $({\mathcal {Z}}\alpha )^7 \log ^2 ({\mathcal {Z}}\alpha )^{-2}$ in the $({\mathcal {Z}} \alpha )$ expansion were included. The uncertainty was estimated assuming that the first neglected higher-order term proportional to $({\mathcal {Z}}\alpha )^7 \log ({\mathcal {Z}}\alpha )^{-2}$ is of natural size. In Ref. [85], it was shown that the previously neglected coefficient $D_{71}$ multiplying the $({\mathcal {Z}}\alpha )^7 \log ({\mathcal {Z}}\alpha )^{-2}$ term is about a factor 16 larger than the coefficient $D_{72}$ multiplying the supposedly more important $({\mathcal {Z}}\alpha )^7 \log ^2 ({\mathcal {Z}}\alpha )^{-2}$ term. This resolved a discrepancy between numerical all-order and analytical $({\mathcal {Z}} \alpha )$-expansion results. Here, we use the all-order result reported in Ref. [86]. The two values in Eq. (D15) are the recoil corrections for point-like and Gaussian distributed nuclear charges, respectively. The uncertainty is due to the latter contribution of the nuclear-finite size. As suggested in Ref. [85], we estimate that the uncertainty of the dimensionless parameter $\delta _\text {fns}P$ due to the values of the nuclear radii is given by , where for $\delta R$ we take the difference between the proton and deuteron radii in the Gauss nuclear model and the experimental values from Eqs. (59) and (2). Furthermore, we include an uncertainty due to approximations made in the calculation of $\delta _\text {fns}P$ ($19\times 10^{-7}$ for H, $65\times 10^{-7}$ for D) and the difference between the nuclear models in Ref. [85, 86].
6. Radiative recoil corrections:^4^
  D16 $$\begin{aligned} \Delta \nu _6=-5.38(32)\,\text {kHz}\qquad [-5.38(11)\,\text {kHz}]. \end{aligned}$$
  As suggested in Ref. [87], the more conservative uncertainty estimate from CODATA is used [10].
7. Nuclear SE:
  D17 $$\begin{aligned} \Delta \nu _7=2.98(11)\,\text {kHz}\qquad [2.98(10)\,\text {kHz}]. \end{aligned}$$
8. Muonic and hadronic VP:
  D18 $$\begin{aligned} \Delta \nu _8=0.006\,\text {kHz}\qquad [0.006\,\text {kHz}]. \end{aligned}$$
  While the numerical value of the sum of muonic and hadronic VP contributions remains unchanged, it has in fact been improved, including in addition the effect of NLO hadronic VP and the muonic VP correction to the electromagnetic electron vertex [55]. We checked that the scatter between theoretical predictions and experimental extraction of the hadronic VP contribution to the muon anomalous magnetic moment $a_\mu$, used as input in Ref. [55], agrees with an updated value based on the new experimental result from Fermilab [88] and the presently recommended Standard Model prediction collected in the Theory Initiative White Paper [89]. It also covers the recent lattice QCD prediction from the BMW Collaboration [90]. The further improvement in precision of the scatter is not relevant at the level of precision required for the isotope shift.
9. Nuclear-polarizability correction:
  D19 $$\begin{aligned} \Delta \nu _{9}= & {} \left[ 18.489 - 0.769\,l_1^{C0_S} \right] \textrm{kHz}\nonumber \\ {}= & {} 18.490(205)\,\text {kHz}\qquad [18.64(2)\,\text {kHz}], \end{aligned}$$
  from combining our results in Eqs. (51) and (55). The uncertainty is dominated by $\Delta \nu _{9}^\textrm{D}$. The decrease of the central value is mainly due to our new EFT prediction of the inelastic contribution to the 2$\upgamma$ exchange in D. The error estimate in Ref. [45] seems to be too low by about a factor of 4; based on Eqs. (17) and (18) from Ref. [48], one should get $0.08~\textrm{kHz}$ instead of 0.02 kHz. In addition, we think that the previously used work [49] might have underestimated the uncertainty. The error estimate presented here is a conservative choice, given the smallness of higher-order terms (in particular, the effect of single-nucleon contributions). We also do not take into account the correlation between the proton contributions in H and D, which would lead to a reduction of the uncertainty estimate.
10. Compensation of the Darwin–Foldy term for the deuteron:
  D20 $$\begin{aligned} \Delta \nu _{10}=11.37\,\text {kHz}\qquad [11.37\,\text {kHz}]. \end{aligned}$$

The total contribution to the isotope shift coming from this Set (ii) is given by:

D21 $$\begin{aligned} \Delta f_\textrm{ii}&=\left[ -4502.48 - 0.769\,l_1^{C0_S} \right] ~\textrm{kHz} \nonumber \\&= -4502.48(40)~\textrm{kHz}\qquad [-4502.66(60)~\textrm{kHz}], \end{aligned}$$

where we added the above uncertainties quadratically.

**Table 9:** Individual contributions to the $2S{-}1S$ transition in H with $R_\infty c$ from Eq. (E4)

Contribution	Value (uncertainty) in Hz	
Dirac eigenvalue	$\Delta f^\textrm{H}_\textrm{i} =2\,466\,068\,540\,936\,672$	$(2\,026)$
One-loop SE and electronic VP	$\Delta \nu ^\textrm{H}_1=-7\,124\,094\,961$	(1)
Two-loop SE, electronic VP, and combined effects	$\Delta \nu ^\textrm{H}_2=-636\,881$	$(1\,733)$
Three-loop SE, electronic VP, and combined effects	$\Delta \nu ^\textrm{H}_3=-1\,509$	(370)
Salpeter recoil correction	$\Delta \nu ^\textrm{H}_4=-2\,068\,223$	
Higher-order pure recoil corrections	$\Delta \nu ^\textrm{H}_5=6\,354$	(7)
Radiative recoil corrections	$\Delta \nu ^\textrm{H}_6=10\,781$	(74)
Nuclear SE	$\Delta \nu ^\textrm{H}_7=-4\,034$	(141)
Muonic and hadronic VP	$\Delta \nu ^\textrm{H}_8=7\,410$	(70)
Nuclear polarizability correction	$\Delta \nu ^\textrm{H}_9=41$	(16)
Subtotal: Lamb shift contributions	$\Delta f^\textrm{H}_\textrm{ii} =-7\,126\,781\,023$	$(1\,780)$
Elastic 2$\upgamma$	$\Delta \nu ^\textrm{H}_{(b)}=35$	(6)
Relativistic higher-order corrections	$\Delta \nu ^\textrm{H}_{(c)}=-928$	(344)
SE contribution to the nuclear size correction	$\Delta \nu ^\textrm{H}_{(d)}=217.0\left( \frac{r_p}{\textrm{fm}}\right) ^2$	
Electronic VP contribution to the nuclear size correction	$\Delta \nu ^\textrm{H}_{(e)}=-54.7\left( \frac{r_p}{\textrm{fm}}\right) ^2$	
Subtotal: higher-order nuclear-size correction	$\Delta \nu ^\textrm{H}_{(b)}+\Delta \nu ^\textrm{H}_{(c)} =-893$	(344)
	$$\Delta \nu ^\textrm{H}_{(d)}+\Delta \nu ^\textrm{H}_{(e)} =162.3\left( \frac{r_p}{\textrm{fm}}\right) ^2$$	
Leading non-relativistic nuclear-size correction	$\Delta f^\textrm{H}_\textrm{iv} =-1\,368\,229 \left( \frac{r_p}{\textrm{fm}}\right) ^2$	

Finally, the smallest correction comes from Set (iii) in Ref. [45]. In the following, we will split the set and separate the contributions that we deduce directly by scaling the leading non-relativistic nuclear-size correction:

D22 $$\begin{aligned} \Delta f_\textrm{iv} = -\frac{7}{24\pi }\frac{\alpha ^4 m_e^3 c^4}{\hbar ^3}\left[ \frac{r_d^2}{(1+m_e/M_d)^3}-\frac{r_p^2}{(1+m_e/M_p)^3}\right] . \end{aligned}$$

The higher-order nuclear-size corrections of Set (iii) are:

(b) Elastic contribution to the 2$\upgamma$ exchange:
  D23 $$\begin{aligned} \Delta \nu _{(b)}&= \left[ 0.590 - 1.607\,l_1^{C0_S} \right] \textrm{kHz}\nonumber \\&=0.587(9)\,\text {kHz}\qquad [0.472\,\text {kHz}], \end{aligned}$$
  from combining our results in Eqs. (52) and (54). The previous result only included the dominant Friar-radius correction.
(c) Relativistic higher-order corrections:
  D24 $$\begin{aligned} \Delta \nu _{(c)} = -2.029(41)\, \textrm{kHz} \qquad [-2.828\,\text {kHz}]. \end{aligned}$$
  Here, we use the recent update from Ref. [87], including the $3\gamma$-exchange effects due to finite nuclear size.
(d) SE contribution to the nuclear size correction:
  D25 $$\begin{aligned} \Delta \nu _{(d)}&=\alpha ^2 \left[ 4 \ln 2 -\frac{23}{4}\right] \Delta f_\textrm{iv} \nonumber \\&=0.830 \, \textrm{kHz} \qquad [0.828\,\text {kHz}]. \end{aligned}$$
  To illustrate the numerical size of the effect, we are using the proton charge radius from the $\mu$H Lamb shift, $r_p(\mu \text {H})$ in Eq. (59), and the CODATA 2018 recommended deuteron charge radius, $r_d(\text {CODATA '18})$ in Eq. (3).
(e) Electronic VP contribution to the nuclear size correction:
  D26 $$\begin{aligned} \Delta \nu _{(e)} =\frac{3\alpha ^2}{4}\,\Delta f_\textrm{iv}= -0.209\, \textrm{kHz} \qquad [-0.209\,\text {kHz}]. \end{aligned}$$

In total, Set (iii) amounts to:

D27a $$\begin{aligned} \Delta f_\textrm{iii}= & {} \Delta \nu _{(b)}+\Delta \nu _{(c)}+\Delta \nu _{(d)}+\Delta \nu _{(e)} \nonumber \\= & {} -0.821(42)~\textrm{kHz}\qquad [ -1.73~\textrm{kHz}], \end{aligned}$$

with

D27b $$\begin{aligned}{} & {} \Delta \nu _{(b)}+\Delta \nu _{(c)}=\left[ -1.439 + 1.607\,l_1^{C0_S} \right] \,\,\textrm{kHz} \nonumber \\{} & {} \qquad \qquad \qquad \quad =\! -1.442(42)~\textrm{kHz}\quad [ -2.356~\textrm{kHz}], \end{aligned}$$

D27c $$\begin{aligned}{} & {} \Delta \nu _{(d)}+\Delta \nu _{(e)} =\alpha ^2 \left[ 4 \ln 2 -5\right] \,\Delta f_\textrm{iv} \nonumber \\{} & {} \qquad \qquad \qquad \quad =0.621 \, \textrm{kHz}\,\,\,\, [0.619\,\text {kHz}]. \end{aligned}$$

Here, we again assumed $r_p(\mu \text {H})$ and $r_d(\text {CODATA '18})$ to illustrate the numerical size of the effect.

Collecting Eqs. (D2), (D21), (D27) and (D22), our final result for the theoretical prediction of the $2S{-}1S$ H–D isotope shift reads:

D28 $$\begin{aligned} f_\textrm{iso}= & {} \Delta f_\textrm{i}+\Delta f_\textrm{ii}+\Delta f_\textrm{iii}+\Delta f_\textrm{iv}\nonumber \\= & {} \Bigg [671\,000\,534.811(411)(828)+ 0.838 \,l_1^{C0_S} \nonumber \\{} & {} -1369.346\, \left( \frac{r_d}{\textrm{fm}}\right) ^2\Bigg ]~ \textrm{kHz}, \end{aligned}$$

where the first uncertainty is the combined uncertainty of $\Delta f_\textrm{i}$–$\Delta f_\textrm{iii}$, and the second uncertainty is due to $r_p(\mu \text {H})$ in $\Delta f_\textrm{iv}$.

## Appendix E: Rydberg constant from $2S{-}1S$ transition in hydrogen and muonic hydrogen Lamb shift

Analogously to Appendix D, we study the *S*-level transition in H:

**Table 10:** Individual contributions to the $2S{-}1S$ transition in D with $R_\infty c$ from Eq. (E4)

Contribution	Value (uncertainty) in Hz	
Dirac eigenvalue	$\Delta f^\textrm{D}_\textrm{i} =2\,466\,739\,545\,007\,700$	$(2\,025)$
One-loop SE and electronic VP	$\Delta \nu ^\textrm{D}_1=-7\,129\,653\,960$	(1)
Two-loop SE, electronic VP, and combined effects	$\Delta \nu ^\textrm{D}_2=-637\,402$	$(1\,734)$
Three-loop SE, electronic VP, and combined effects	$\Delta \nu ^\textrm{D}_3=-1\,510$	(370)
Salpeter recoil correction	$\Delta \nu ^\textrm{D}_4=-1\,035\,575$	
Higher-order pure recoil corrections	$\Delta \nu ^\textrm{D}_5=3\,295$	(3)
Radiative recoil corrections	$\Delta \nu ^\textrm{D}_6=5\,398$	(37)
Nuclear SE	$\Delta \nu ^\textrm{D}_7=-1\,059$	(35)
Muonic and hadronic VP	$\Delta \nu ^\textrm{D}_8=7\,416$	(70)
Nuclear polarizability correction	$\Delta \nu ^\textrm{D}_9=18\,531$	(204)
Compensation of the Darwin–Foldy term for the deuteron	$\Delta \nu ^\textrm{D}_{10}=11\,369$	
Subtotal: Lamb shift contributions	$\Delta f^\textrm{D}_\textrm{ii} =-7\,131\,283\,498$	$(1\,787)$
Elastic 2$\upgamma$	$\Delta \nu ^\textrm{D}_{(b)}=622$	(7)
Relativistic higher-order corrections	$\Delta \nu ^\textrm{D}_{(c)}=-2\,961$	(357)
SE contribution to the nuclear size correction	$\Delta \nu ^\textrm{D}_{(d)}=217.1\left( \frac{r_d}{\textrm{fm}}\right) ^2$	
Electronic VP contribution to the nuclear size correction	$\Delta \nu ^\textrm{D}_{(e)}=-54.7\left( \frac{r_d}{\textrm{fm}}\right) ^2$	
Subtotal: higher-order nuclear-size correction	$\Delta \nu ^\textrm{D}_{(b)}+\Delta \nu ^\textrm{D}_{(c)} =-2\,340$	(357)
	$$\Delta \nu ^\textrm{D}_{(d)}+\Delta \nu ^\textrm{D}_{(e)} =162.4\left( \frac{r_d}{\textrm{fm}}\right) ^2$$	
Leading non-relativistic nuclear-size correction	$\Delta f^\textrm{D}_\textrm{iv} =-1\,369\,346 \left( \frac{r_d}{\textrm{fm}}\right) ^2$	

E1 $$\begin{aligned} E^\text {H} _{2S{-}1S}=h \, f^\text {H}_{2S{-}1S}. \end{aligned}$$

The individual contributions are listed in Table 9. In total, we find:

E2 $$\begin{aligned} f^\textrm{H}_{2S{-}1S}= & {} \Delta f^\textrm{H}_\textrm{i}+\Delta f^\textrm{H}_\textrm{ii}+\Delta f^\textrm{H}_\textrm{iii}+\Delta f^\textrm{H}_\textrm{iv}\nonumber \\= & {} \left[ 0.74960091418756\,\frac{R_\infty c}{\textrm{Hz}}-7\,126\,781\,916(1\,813)\right. \nonumber \\{} & {} \left. -1\,368\,229\left( \frac{r_p}{\textrm{fm}}\right) ^2\right] \,\textrm{Hz}. \end{aligned}$$

If not specified differently in Appendix D, we follow the CODATA procedure for the error estimate [10]. For $\Delta f_i$, we use the exact formula to deduce the uncertainty, see Ref. [45, Sec. III C.]. We can use Eq. (E2) and $r_p(\mu \text {H})$ to extract the Rydberg constant from the measured transition [91]:

E3 $$\begin{aligned} f^\textrm{H}_{2S{-}1S}=2\,466\,061\,413\,187\,035(10)\,\textrm{Hz}. \end{aligned}$$

Our result:

E4 $$\begin{aligned} R_\infty c=3.289\,841\,960\,2509(27)\times 10^{15}\,\textrm{Hz}, \end{aligned}$$

is in perfect agreement with Ref. [8, Eq. (22)] and Eq. (D1). Compared to the latter, the uncertainty is more than a factor 2 better. We can use this value to extract the deuteron radius from the $2S{-}1S$ transition in D, since it is largely driven by the measured Lamb shift in $\mu$H.

## Appendix F: $2S{-}1S$ transition in deuterium

Analogously to Appendix E, we study the *S*-level transition in D:

F1 $$\begin{aligned} E^\text {D}_{2S{-}1S}=h \, f^\text {D}_{2S{-}1S}. \end{aligned}$$

The individual contributions are listed in Table 10. For the $3\gamma$-exchange contribution, we use Ref. [87, Eq. (104)] with $r_d(\mu \text {D})$ from Eq. (4) and apply $10\,\%$ uncertainty for the inelastic part and $100\,\%$ for the single-nucleon part, where for the latter we also assume a correlation between 1*S* and 2*S* levels. The updated theory prediction for the $2S{-}1S$ transition in D, including the 2$\upgamma$ exchange from EFT, then reads:

F2 $$\begin{aligned} f^\textrm{D}_{2S{-}1S}= & {} \Delta f^\textrm{D}_\textrm{i}+\Delta f^\textrm{D}_\textrm{ii}+\Delta f^\textrm{D}_\textrm{iii}+\Delta f^\textrm{D}_\textrm{iv}\nonumber \\= & {} \left[ 2\,466\,732\,413\,721\,862(2\,724)-1\,369\,346\left( \frac{r_d}{\textrm{fm}}\right) ^2\right] \,\textrm{Hz}. \nonumber \\ \end{aligned}$$

This is used in Sect. 6.1 to extract the deuteron charge radius.

## Appendix G: Neutron charge radius and deuteron structure radius

Another interesting quantity is the deuteron structure radius, defined as:

G1 $$\begin{aligned} r_\textrm{str}^2=r_d^2-2\,r_0^2. \end{aligned}$$

This definition implies that the difference of squared deuteron and proton charge radii is related to the sum of squared deuteron structure radius and neutron charge radius:

G2 $$\begin{aligned} r_d^2-r_p^2=r_\textrm{str}^2+\frac{3}{4M_p^2} + r_n^2. \end{aligned}$$

The deuteron structure radius has recently been predicted in $\chi$ET [27]:

G3 $$\begin{aligned} r_\textrm{str}(\chi \textrm{ET})=1.9729\left( {}^{+15}_{-12}\right) \,\textrm{fm}. \end{aligned}$$

We can use this value as an alternative reference to fix the unknown LEC from Eq. (15):

G4 $$\begin{aligned} l_1^{C0_S}=-2.41\left( {}^{+32}_{-26}\right) (35)\times 10^{-3}=-2.41\left( {}^{+48}_{-44}\right) \times 10^{-3}, \end{aligned}$$

where the first error is due to $r_\textrm{str}$ and the second is due to *Z*. Using in addition the $2S{-}1S$ H–D isotope shift in Eq. (D28), we extract the neutron charge radius as:

G5 $$\begin{aligned} r_n^2=-0.105\left( {}^{+5}_{-6}\right) \,\textrm{fm}^2, \end{aligned}$$

in exact agreement with Ref. [27], but in slight disagreement with the value used by us, see Eq. (31). We checked that the effect of the nucleon charge radii entering our EFT prediction of the 2$\upgamma$ exchange adds up to 0.021 kHz. Since the size of these nucleon-radius contributions is covered by the uncertainty budget in Eq. (58), we can safely ignore this correlation in the above extraction of $r_n$. Note that even though the value of $l_1^{C0_S}$ in Eq. (G3) agrees well with Eq. (16), the corresponding values for $r_d$ disagree, since it also depends on $r_n$ through $r_0$, cf. Eq. (15).

## Footnotes

1 Note that the leading pion-nucleon loop contribution to the subtraction-function correction was previously calculated with an approximate formula [39, Eq. (17a)] and has been updated here.

2 Note that in the final sum of radius-dependent terms in Ref. [5, Table 2] the entry #r8 has been included with a wrong sign.
